# Extracellular DNA and Type IV Pilus Expression Regulate the Structure and Kinetics of Biofilm Formation by Nontypeable *Haemophilus influenzae*

**DOI:** 10.1128/mBio.01466-17

**Published:** 2017-12-19

**Authors:** Jayajit Das, Elaine Mokrzan, Vinal Lakhani, Lucia Rosas, Joseph A. Jurcisek, William C. Ray, Lauren O. Bakaletz

**Affiliations:** a Battelle Center for Mathematical Medicine, The Research Institute at the Nationwide Children's Hospital, Columbus, Ohio, USA; b Center for Microbial Pathogenesis, The Research Institute at the Nationwide Children’s Hospital, Columbus, Ohio, USA; c Department of Pediatrics, the Ohio State University College of Medicine, Columbus, Ohio, USA; d Department of Biophysics Graduate Program, the Ohio State University, Columbus, Ohio, USA; University of Washington

**Keywords:** agent-based modeling, biofilms, nontypeable *Haemophilus influenzae*, otitis media, pair correlation

## Abstract

Biofilms formed in the middle ear by nontypeable *Haemophilus influenzae* (NTHI) are central to the chronicity, recurrence, and refractive nature of otitis media (OM). However, mechanisms that underlie the emergence of specific NTHI biofilm structures are unclear. We combined computational analysis tools and *in silico* modeling rooted in statistical physics with confocal imaging of NTHI biofilms formed *in vitro* during static culture in order to identify mechanisms that give rise to distinguishing morphological features. Our analysis of confocal images of biofilms formed by NTHI strain 86-028NP using pair correlations of local bacterial densities within sequential planes parallel to the substrate showed the presence of fractal structures of short length scales (≤10 μm). The *in silico* modeling revealed that extracellular DNA (eDNA) and type IV pilus (Tfp) expression played important roles in giving rise to the fractal structures and allowed us to predict a substantial reduction of these structures for an isogenic mutant (Δ*comE*) that was significantly compromised in its ability to release eDNA into the biofilm matrix and had impaired Tfp function. This prediction was confirmed by analysis of confocal images of *in vitro* Δ*comE* strain biofilms. The fractal structures potentially generate niches for NTHI survival in the hostile middle ear microenvironment by dramatically increasing the contact area of the biofilm with the surrounding environment, facilitating nutrient exchange, and by generating spatial positive feedback to quorum signaling.

## IMPORTANCE

NTHI is a major bacterial pathogen for OM, which is a common ear infection in children worldwide. Chronic OM is associated with bacterial biofilm formation in the middle ear; therefore, knowledge of the mechanisms that underlie NTHI biofilm formation is important for the development of therapeutic strategies for NTHI-associated OM. Our combined approach using confocal imaging of NTHI biofilms formed *in vitro* and mathematical tools for analysis of pairwise density correlations and agent-based modeling revealed that eDNA and Tfp expression were important factors in the development of fractal structures in NTHI biofilms. These structures may help NTHI survive in hostile environments, such as the middle ear. Our *in silico* model can be used in combination with laboratory or animal modeling studies to further define the mechanisms that underlie NTHI biofilm development during OM and thereby guide the rational design of, and optimize time and cost for, benchwork and preclinical studies.

## INTRODUCTION

Nontypeable *Haemophilus influenzae* (NTHI) is a predominant bacterial pathogen of otitis media (OM), a common middle ear infection for children worldwide under the age of 15 years (1). Chronic and recurrent OM are both characterized by the formation of biofilms in the middle ear, often by NTHI (1, 2). Biofilms are three-dimensional complex structures formed by bacteria and are usually associated with either abiotic or biotic substrates, including mucosal surfaces (3). Biofilm formation contributes to bacterial resistance to host immune responses or antibiotics. Distinct spatial patterns, such as tower or mushroom structures, arise in biofilms formed by different bacterial species under specific experimental conditions. Such spatial patterns can be associated with survival of the bacteria within the biofilm. For example, mushroom-like structures separated by water-filled regions occurred in biofilms formed by *Pseudomonas aeruginosa* in flow chambers when glucose was used as a carbon source in the medium (4). The mushroom structures were proposed to create a circulatory system for efficient nutrient supply and waste removal (5, 6). Similar morphological features may promote NTHI survival in the hostile host environment. Therefore, characterization of specific spatial patterns in NTHI biofilms and uncovering the mechanisms that give rise to those morphological features are important for understanding the establishment and pathogenesis of OM and for developing therapeutic strategies for treating OM.

Recent studies have identified several factors that play important roles in regulating the stability and morphology of NTHI biofilms (7–17). One such key factor is extracellular DNA (eDNA) (8, 11), a major structural component of the extracellular polymeric substance (EPS) that comprises the biofilm matrix. The eDNA within an NTHI biofilm forms a tight meshwork of criss-crossing eDNA strands (9, 11), which is stabilized by DNABII proteins bound to the vertices of crossed eDNA strands. The origin of eDNA in bacterial biofilms remains unclear, but it is often attributed to bacterial autolysis (18) or release of outer membrane vesicles. However, a recent study by Jurcisek et al. (19) described a novel mechanism: DNA and DNABII protein release occurs via a newly identified inner membrane pore complex (TraC and TraG) with homology to type IV secretion-like systems and through the ComE pore, which is part of the machinery for expression of type IV twitching pili and is located in the bacterial outer membrane. The role of the eDNA network in bacterial cell organization during the process of biofilm formation is not well understood. High-resolution time-lapse microscopy studies of *Pseudomonas aeruginosa* biofilms showed that the eDNA network facilitates mass movement of bacterial cells along “furrows” within the biofilm structure (20). This movement is mediated by type IV pili (Tfp), which execute a repeating pattern of extension, attachment, and retraction that results in the ratchet-like motion termed twitching motility. Mokrzan et al. (13) demonstrated that NTHI Tfp are required for the formation of “tower” structures characteristic of NTHI biofilms. Taken together, these data suggest that both the eDNA network and Tfp expression play important roles in NTHI biofilm structural development.

However, a quantitative characterization of spatial patterns found within biofilms formed by NTHI and its mutant strains, and how these patterns are regulated by the eDNA network and Tfp expression, are not well understood. We addressed the above issues by combining data analysis approaches rooted in statistical physics (21–23), confocal microscopy imaging of NTHI biofilms formed *in vitro*, and *in silico* mathematical modeling (24, 25). Our analysis of the confocal images of biofilms formed *in vitro* by wild-type (WT) NTHI revealed formation of fractal structures in the organization of wild-type NTHI cells within the biofilms at short distances (≤10 μm). Emergence of fractal structures could potentially help bacteria survive in a hostile environment (e.g., the middle ear) by facilitating nutrient absorption or by locally trapping molecules that mediate quorum sensing. The agent-based *in silico* model showed that the emergence of such structures in wild-type NTHI biofilms is facilitated by the eDNA network and Tfp function. This was further confirmed by the agreement of model predictions regarding the large reduction in these fractal structures in biofilms formed *in vitro* by a Δ*comE* mutant strain of NTHI. The Δ*comE* mutant has impaired Tfp function, and the biofilms formed by the Δ*comE* strain *in vitro* contain substantially less eDNA than those formed by the wild type. The developed *in silico* model can be used to test various hypotheses regarding biofilm formation by mutant NTHI strains and perturbation of biofilm structures by different drugs.

## RESULTS

### Analysis of the pair correlation function in bacterial density reveals the presence of fractal structures of smaller length scales (≤10 μm) in older (≥40 h) NTHI biofilms formed *in vitro*.

**FIG 1:**
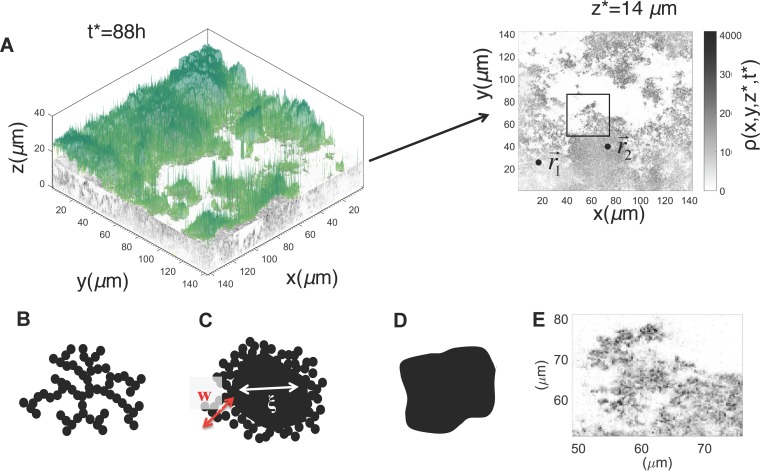
Analysis of confocal microscopy imaging data obtained for *in vitro* NTHI biofilms. (A, left) The three-dimensional density profile [ρ(*x*,*y*,*z*,t*)] for NTHI from the confocal microscopy imaging data, which were obtained from a sample of an *in vitro*-grown NTHI biofilm at t* = 88 h. The NTHI density at each *z* plane is shown in grayscale, with darker intensities indicating higher densities. The biofilm surface is shown in color. (Right) NTHI density ρ(*x*,*y*,z*,t*) in a *z* plane (e.g., z* = 14 μm) was analyzed by computing the pair correlation between a pair of points (${\overset{\to }{r}}_{1}$ and ${\overset{\to }{r}}_{2}$) in the *z* plane. (B to D) Schematic depiction of examples where the bacterial cells were organized in a mass fractal (B), a surface fractal where a solid cluster contained an interface with fractal structures (C), or a solid cluster with a smooth interface (D). (E) Spatial organization of NTHI cells in the *z* plane at z* = 14 μm, shown as a magnification of the area enclosed in the black box in panel A, right panel.

**FIG 2:**
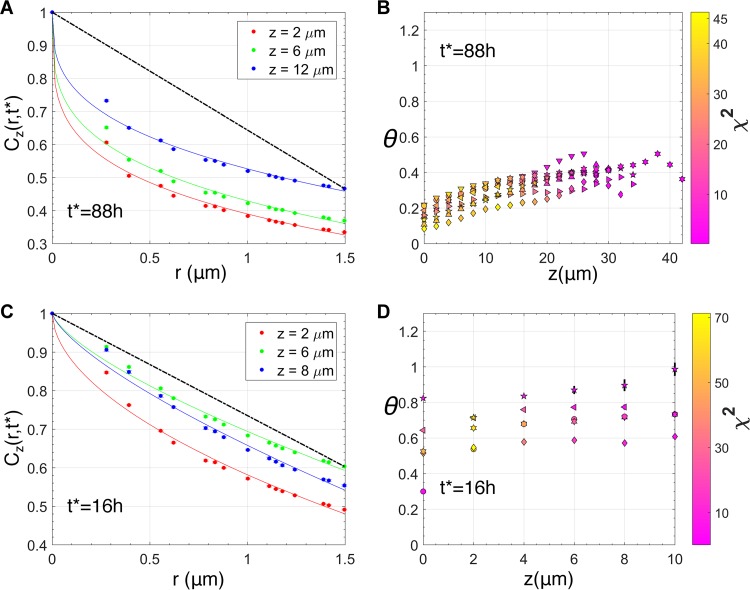
Surface fractal structures in NTHI biofilms. (A) Variation of *C*_*z*_(*r*,t*)/*C*_*z*_(*r* = 0,t*), with *r* at a fixed t* (88 h) and at three different *z* planes within a replicate of the *in vitro* NTHI biofilm. The θ exponents obtained by fitting the function in equation 2 for the data at *z* = 2, 6, and 12 μm were 0.25, 0.28, and 0.33, respectively. The fits are shown with solid lines. The straight line shown in black gives an example for the case with θ = 1. (B) Values of the estimated θ exponents at different *z* stacks, calculated by fitting equation 2 to C_z_(*r*,t*) for *r* ≤1.5 μm, calculated for 8 different replicates for the 88-h NTHI biofilm. The different symbols indicate different replicates. All the estimated values for θ were smaller than 1, indicating the presence of scale-invariant or fractal structures at short length scales. The symbol colors (see the color bar on the right) display the χ^2^ values, which indicate the quality of the fit (equation 3) used to estimate the θ values. A lower χ^2^ value indicates a better fit of the data with equation 3. (C) Variation of *C*_*z*_(*r*,t*)/*C*_*z*_(*r* = 0,t*) with *r* at a fixed t* (16 h) and at three different *z* planes within a replicate of the *in vitro* NTHI biofilm. The estimated θ exponent values at *z* = 2, 6, and 8 μm were 0.54, 0.71, and 0.72, respectively. The solid straight line in black shows the reference for the θ = 1 case. (D) Estimated values for θ at different values of *z* for different replicates of the 16-h biofilm. Unlike the 88-h biofilm, the estimated θ value was closer to 1 for several *z* planes, suggesting the presence of sharp but smooth interfaces in those planes within the biofilm. The visualization scheme is the same as that for panel B.

We analyzed spatial pair correlations between bacterial densities in confocal microscopy images for biofilms formed in static culture by NTHI strain 86-028NP in order to determine the presence of specific spatial patterns in the morphology. The local intensity in the images was assumed to be proportional to the local bacterial density ρ(*x*,*y*,*z*,*t*), where {*x*,*y*,*z*} denotes the spatial location within the biofilm and *t* indicates the age of the biofilm (Fig. 1A). We calculated the pair correlation function *C*_*z*_(*r*,*t*) between the bacterial densities at two locations separated by distance *r* in a plane *x*-*y* parallel to and residing at a fixed distance (*z*) from the substrate (Fig. 1A) (see Materials and Methods for further details on our procedure). The behavior of *C*_*z*_(*r*,*t*) at short distances (*r*), characterized by exponent θ, was used to determine the nature of the spatial patterns for that length scale (see Materials and Methods). The value of exponent θ contains information regarding the nature of the spatial pattern for that length scale (21–23, 26). For example, if the bacterial cluster in the two-dimensional *x*-*y* plane forms a scale-invariant structure, a mass fractal (Fig. 1B) or a surface fractal (a solid structure with a fractal interface) (Fig. 1C), or a solid structure with a sharp nonfractal interface (Fig. 1D), the values of θ will be <0, between 0 and 1, and equal to 1, respectively. Visual inspection of a two-dimensional density profile of the confocal images at a *z* plane for an NTHI biofilm grown for 88 h suggested the presence of fractal structures at the interfaces of the bacterial clusters with the surrounding liquid environment (Fig. 1E). To investigate the presence of such structures, we analyzed *C*_z_(*r*,t*) for the biofilms formed at t* = 16 h, 40 h, 64 h, and 88 h after biofilm growth was initiated. *C*_z_(*r*,t*), calculated for the biofilm at many planes parallel to the substrate at t* = 88 h, could be fitted with θ values smaller than unity for distances (Fig. 2A and B; see also Fig. S1 in the supplemental material), with *r* between 0.27 and 10 μm, indicating the presence of surface fractal structures at shorter distances (*r* ≈ 10 μm). Similar behavior in *C*_*z*_(*r*,t*) was present at other time points (e.g., t* = 40 h, 64 h) as well. At 16 h, most of the bacterial clusters showed the presence of surface fractal structures at many *z* planes (Fig. 2C and D) but also displayed solid structures with sharp but smooth interfaces (θ ≈ 1) (Fig. 2C and D) at several *z* planes. Thus, the analysis showed the presence of fractal interfaces within short length scales (≤10 μm) in older (≥40 h) biofilms.

**FIG 3:**
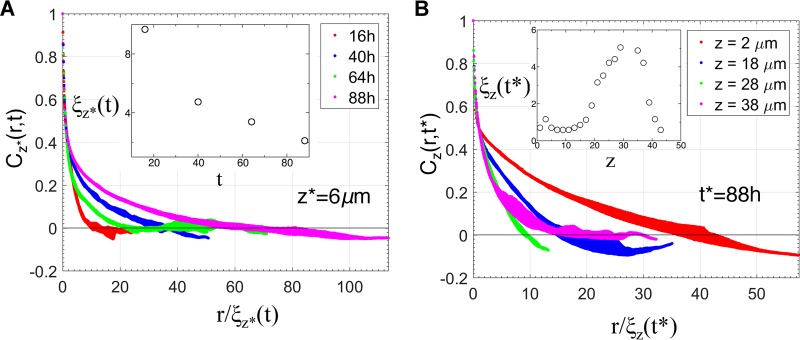
Multiple length and time scales underlie NTHI biofilm growth. (A) Variation of *C*_z*_(*r*,*t*)/*C*_z*_(*r* = 0,t) with *r*/ξ_z*_(*t*) at a fixed z* (6 μm) and at four different times (*t* = 16 h, 40 h, 64 h, and 88 h) for a replicate of the NTHI biofilm formed *in vitro*. ξ_z*_(*t*) at different times was calculated using the equation *C*_z*_[*r* = ξ_z*_(*t*),*t*]/*C*_z*_(*r* = 0,*t*) = 1/2. The presence of a characteristic length scale, ξ_z*_(*t*), would have resulted in an overlap of the different graphs on a single scaling function. The absence of such a behavior indicates the existence of multiple time and length scales in the underlying kinetics. The inset shows the variation of ξ_z*_(*t*) with *t* for the data shown. (B) Variation of *C*_*z*_(*r*,t*)/*C*_*z*_(*r* = 0,t*) with *r*/ξ_*z*_(*t*) at a fixed time (t* = 88 h) and at four different *z* planes (2 μm, 18 μm, 28 μm, and 38 μm) for a replicate of the NTHI biofilm formed *in vitro*. The variation of ξ_*z*_(t*) (in micrometers) with *z* (in micrometers) is shown in the inset. The absence of any scaling in the *C*_*z*_(*r*,t*)/*C*_*z*_(*r* = 0,t*) versus *r*/ξ_*z*_(t*) data indicates the presence of multiple length scales at different *z* planes.

10.1128/mBio.01466-17.2 FIG S1  Different types of fractal structures at different length scales. The C_z_(*r*,t*)/C_z_(0,t*) versus *r* data at t* = 88 h on multiple *z* planes were calculated from *in vitro* WT NTHI biofilms. Data in panels A and B are from different replicates. The fits are shown as solid lines, colored the same as their respective data. (A) From the same slices shown in Fig. 2A, we fit the data with two different exponents and coefficients, as described in equation 4. The first exponent (θ_<_) and coefficient (a_<_) apply to an *r* value of ≤1.5 μm; the second exponent (θ_>_) and coefficient (a_>_) apply to an *r* value between 1.5 and 10 μm. The data can be fitted well for θ_<_ (0.245, 0.28, 0.33) and θ_>_ (0.11, 0.15, 0.22) values smaller than unity. The corresponding coefficients are a_<_ (0.61, 0.57, 0.47) and a_>_ (0.65, 0.6, 0.49). (B) From the same slices shown in Fig. 3B, we fit the data with three different exponents as described in equation 5 in Materials and Methods. The first exponent (θ_1_) and coefficient (a_1_) apply to the range *r* ≤1.5 μm (*r*_1_); the second exponent (θ_2_) and coefficient (a_2_) apply to the range of *r* values between 1.5 and 10 μm (*r*_2_); finally, the third exponent (θ_3_) and coefficient (a_3_) apply to the range *r* ≥10 μm. The data fit well for θ_1_ (0.19, 0.42, 0.505), θ_2_ (0.18, 0.31, 0.29), and θ_3_ (0.24, 0.29, 0.06). The corresponding coefficients were *a*_1_ (0.49, 0.28, 0.36), *a*_2_ (0.46, 0.3, 0.425), and *a*_3_ (0.4, 0.33, 0.79). Panels A and B show that the data can be fitted with different exponents at different length scales. This result indicates there are different types of fractal patterns at small lengths (*r* < 1.5 μm), greater lengths (1.5 μm < *r* < 10 μm), and long lengths (*r* >10 μm). Download FIG S1, PDF file, 0.8 MB. Copyright © 2017 Das et al. 2017 Das et al. This content is distributed under the terms of the Creative Commons Attribution 4.0 International license.

### Existence of multiple time and length scales in the biofilm structures.

Next, we investigated whether there was a characteristic length and time scale associated with the organization of the bacterial cells within the biofilm. The presence of such a behavior points to the relevance of specific processes in determining the spatial structure in the system of interest. For example, theoretical models in statistical physics (27) describing growth of ordered magnetic domains display emergence of a characteristic length and time scale associated with the size of the ordered magnetic domains, where the domain growth is driven by the surface tension of the domains. In the presence of such a characteristic length scale ξ_*z*_(*t*), *C*_z_(*r*,*t*) at a fixed *z* (=z*) but different times (e.g., *t*_1_ and *t*_2_) behaves as *C*_z*_(*r*,*t*_1_)/*C*_z*_(0,*t*_1_) = *f*_*z*_[*r*/ξ_z*_(*t*_1_)] and *C*_z*_(*r*,*t*_2_)/*C*_z*_(0,*t*_2_) = *f*_z*_[*r*/ξ_z*_(*t*_2_)]. Thus, *C*_z*_(*r*,*t*) calculated for density profiles collected at two different times overlaps on the same scaling function *f*_z*_(*x*), where *r* is scaled as *r* → *r*/ξ_z*_(*t*). The correlation length ξ_z*_(*t*) is calculated using the equation, *C*_z*_[*r* = ξ_z*_(*t*),*t*]/*C*_z*_(0,*t*) = 1/2, i.e., ξ_z*_(t) describes a length scale at which the normalized density correlation decreases to 1/2 and can be related to sizes of the NTHI-rich regions in the *z* = z* plane. The analysis of the biofilm structures showed an absence of such a scaling form (Fig. 3A), revealing the existence of multiple length scales associated with different time scales. Next, we checked if the structures at different *z* planes at a fixed time (*t* = t*) contained a characteristic length scale, which would be reflected in a scaling behavior as *C*_*z*_(*r*,t*)/*C*_*z*_(0,t*) = *g*_t*_[*r*/ξ_*z*_(t*)]. In the presence of such scaling, the graphs showing the variation of *C*_*z*_(*r*,t*) with *r* at multiple *z* planes at a fixed time *t* (=t*) will overlap on the same scaling function *g*_t*_(*x*) when *r* is scaled as *r* → *r*/ξ_*z*_(t*). We found an absence of any scaling behavior (Fig. 3B), indicating the presence of multiple length scales at different z planes in the same biofilm. The variation of ξ_*z*_(t*) with z showed a slow increase as the distance from the substrate increased and then a slight decrease near the top of the biofilms (Fig. 3B, inset). This behavior revealed the presence of a denser organization (bacterial cluster size of ≤1 μm, void size of ≤1 μm) of the bacteria near the substrate that segued into structures with larger clusters (≥5 μm) of bacteria separated by larger voids (≥20 μm) at intermediate distances (~30 μm) from the substrate.

### A predictive *in silico* model describes biofilm formation *in vitro* by wild-type NTHI.

The above analysis of the *in vitro* NTHI biofilm morphologies showed the presence of specific spatial patterns (e.g., surface fractal structures) and the absence of any characteristic length scale, implying that the multiple processes spanning a range of time and length scales need to be considered when developing an *in silico* model for describing biofilm formation. We detail below a coarse-grained agent-based model that we were able to use to describe qualitatively the emergence of the above morphological patterns in the biofilms formed by wild-type NTHI and by a mutant strain of NTHI.

**FIG 4:**
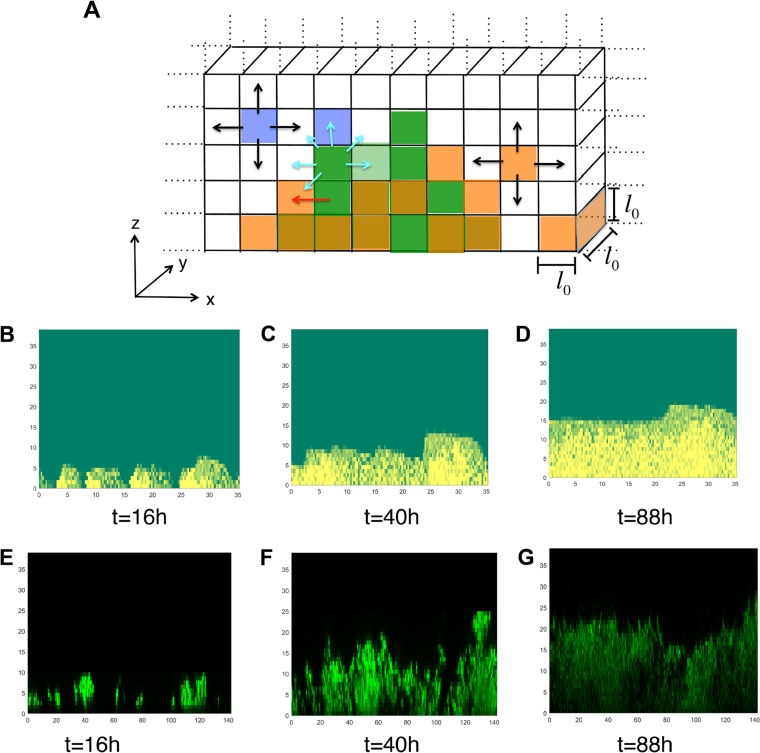
Profiles of biofilms generated in the *in silico* model. (A) Schematic diagram showing the compartments in the *in silico* model. The colored compartments display a typical configuration in the simulation. The compartments occupied with *n*_thres_ or less than *n*_thres_ NTHI bacterial cells are shown in dark or light green, respectively. The compartments occupied with eDNA strands are shown in orange, and the violet compartments denote compartments occupied by planktonic NTHI bacterial cells. The compartments where NTHI and eDNA coexisted appear in dark orange. The unit of length in all the figures are in μm. The possible movements corresponding to diffusion, mass movements, and twitching movement via Tfp are shown with black, red, and cyan arrows. (B to D) Organization of biofilm-associated NTHI bacteria in the *in silico* model in the *x-z* plane at *t* = 16 h (B), *t* = 40 h (C), and *t* = 88 h (D). The substrate lies along the *z* = 0 line. The value for NTHI in each compartment is shown using a color map, where yellow and green indicate high and low numbers, respectively. (E to G) The organization of the NTHI bacterial cells in the confocal images of NTHI biofilms formed *in vitro* are shown in the *x-z* plane for the same times, *t* = 16 h (E), *t* = 40 h (F), and *t* = 88 h (G) for a qualitative comparison.

The agent-based model described the biofilm growth on a quasi-two-dimensional simulation box representing biofilm growth in the *x*-*z* plane on a substrate that lies in the *z* = 0 line (Fig. 4A). The simulation box of size *L*_*x*_ (=128 μm) by *L*_*z*_ (=40 μm) by *l*_0_ (=1 μm) is discretized on a regular lattice composed of small cubic compartments of size *l*_0_ by *l*_0_ by *l*_0_ (*l*_0_ = 1 μm) (Fig. 4A). The model describes the biofilm growth in terms of a set of agents, namely, NTHI bacterial cells associated with the biofilm, planktonic NTHI bacterial cells, eDNA strands, and nutrients. These agents change in time following a set of rules that represent key processes involved in NTHI biofilm growth *in vitro*. The time evolution in the model is implemented using a kinetic Monte Carlo scheme (25). The details of the simulation method are provided in Materials and Methods and Text S1, and details regarding the parameter values, model assumptions, and their connection with known experimental results are shown in Table 1 and also in the supplemental material. Briefly, processes that were modeled in the simulation were the following. (i) For modeling NTHI replication and movement in the biofilm, individual NTHI bacterial cells residing in a compartment consume nutrients within the compartment and replicate at a given rate. The replication of NTHI ceases if the nutrient concentration in a compartment falls below a threshold, *c*_thres_ (=1 molecule/μm^3^). When the number of biofilm-associated NTHI cells in a compartment exceeds a maximum packing number (*n*_thres_), the excess bacteria are moved to an adjacent compartment that can accommodate the excess bacteria without exceeding the threshold *n*_thres_ (Fig. 4A). This rule represents passive movement of NTHI due to the mechanical forces exerted by the neighboring bacteria in a tightly packed region. (ii) For modeling development of the eDNA network, eDNA strands can be present in the compartments. The eDNA strands are produced by a small fraction of NTHI cells at a fixed rate. The simulation is started with a small amount of eDNA present in the system. The eDNA strands that are in contact with the substrate become immediately connected to the substrate and initiate the formation of an eDNA network. The substrate-bound eDNA strands can irreversibly bind to eDNA strands in neighboring compartments and spread the network spatially. The eDNA strands in the eDNA network are considered immobile, and eDNA strands that are not connected to the eDNA network can diffuse into the neighboring compartments (Fig. 4A). Exact mechanisms behind the formation of the eDNA network are not known. DNABII proteins play a role in stabilizing the eDNA network (8); however, precise mechanisms for this stabilization are not clear. This rule is used to minimally develop an eDNA network in the model. (iii) To model NTHI dispersion, we consider that NTHI bacterial cells in the biofilm can disperse into the surrounding liquid medium. We hypothesized that the rate of dispersion is lower in a compartment that is a part of the eDNA network (Table 1). (iv) For modeling movement of NTHI within the eDNA network, we hypothesized that the NTHI bacterial cells move between adjacent compartments when the compartments belong to the eDNA network (Fig. 4A). This process represents a potential Tfp-driven movement of NTHI on eDNA strands. (v) To model nutrient injection and diffusion, a nutrient is introduced into the system twice in 24 h following the 16 h plus 8 h schedule used in the experiments. The nutrient diffusion is much faster than the other rates considered in the model. Therefore, we homogenized the nutrient concentration spatially at every time step in the simulation (see Text S1 for more details). (vi) In modeling diffusion of planktonic NTHI cells, the NTHI bacterial cells in the liquid medium are considered to be in the planktonic state and to move between the compartments via Brownian motion (Fig. 4A). (vii) For modeling removal of planktonic bacteria, we removed 80% of the planktonic bacteria each time the nutrient was added in the simulation. This was done to mimic the step in the experimental protocol where the older medium was removed by aspiration and replaced by fresh medium. Removal of the medium also removed the bacteria in the supernatant from the culture well. Values for the rates and other parameters used in the modeling, as well as the supporting references from the literature, are provided in Table 1.

10.1128/mBio.01466-17.1 TEXT S1  Calculations for the parameters used in the *in silico* model. Download TEXT S1, PDF file, 0.1 MB. Copyright © 2017 Das et al. 2017 Das et al. This content is distributed under the terms of the Creative Commons Attribution 4.0 International license.

**TABLE 1:** Rules, parameters, and parameter values used in the agent-based model

Rule	Biofilm process	Rule implemented in model	Parameter value and rate (per min)	Comment(s)
1	NTHI replication	*N*_NTHI_ and concentration of nutrient (N_c_) in a compartment increases and decreases by 1, respectively (*N*_NTHI_→ *N*_NTHI_ + 1; *N*_c_ → *N*_c_ – 1)	NTHI replication rate (*k*_repli_) of 0.0167 NTHI cells/min (*k*_1_ = *k*_repli_ × N_NTHI_ × N_c_)	Based on 1 division/60 min (46); for slowdown of growth rate due to biofilm aging, we assumed replication rate in NHTI biofilm decreased by ½ after 3 days
2	eDNA production	No. of eDNA strands (*N*_eDNA_) in a compartment increases by 1 (*N*_NTHI_ → *N*_NTHI_ + 1) at rate of *k*_dnaprod_ at every MC step	*k*_dnaprod_ = 0.003 molecules/min until 72 h (*k*_2_ = *k*_dnaprod_)	Rate was calculated using measurements in reference 47; see also Text S1
3	eDNA diffusion	eDNA strands in a compartment but not attached to eDNA network move to nearest neighboring compartments with diffusion rate of *D*_eDNA_	We used *D*_eDNA_ value of 10 μm^2^/min (*k*_3_ = *D*_eDNA_/*l*_0_^2^ × *N*_eDNA_; *N*_eDNA_ ≡ no. of eDNA in compartment)	D_eDNA_ calculation assumes eDNA strands are 1.8 × 10^4^ bp (see Text S1 for details)
4	Diffusion of planktonic NTHI	Planktonic NTHI (pNTHI) in a compartment diffuse to nearest neighboring compartments with diffusion rate *D*_pNTHI_	We used *D*_pNTHI_ of 10 μm^2^/min (*k*_4_ = *D*_pNTHI_/*l*_0_^2^ × *N*_pNTHI_; *N*_pNTHI_ is the no. of pNTHI cells in a compartment)	*D*_pNTHI_ calculation assumes Stokes-Einstein formula (48); see Text S1 for details
5	NTHI dispersion	NTHI in biofilm in a compartment (*N*_NTHI_) disperses to supernatant in same compartment with rate of *k*_dispers_; if compartment is part of eDNA network, rate is 5 times smaller	*k*_dispers_ is taken to be 0.001 molecules/min [*k*_5_ = *k*_dispers_ × (*N*_NTHI_)^2^]	NTHI disperses into supernatant; AI-2-induced quorum sensing along with Tfp appear to regulate this effect (34); we assumed density-dependent rate to represent positive feedback in quorum sensing (49); we hypothesized that dispersion rate is lower when a compartment is part of eDNA network; this can arise due to adherence of NTHI to eDNA by Tfp as well as trapping NTHI via encasement created by eDNA network; we also tested a variant of the model where NTHI dispersion occurred at higher rates in compartments farther from substrate at *z* = 0; results were qualitatively similar to our model (Fig. S7)
6	eDNA binding	eDNA network-bound strands in a compartment bind free eDNA strands in adjacent compartments that are not part of the eDNA network	*k*_dnastick_ = 0.003 [*k*_6_ = *k*_dnastick_ × *N*_eDNA(network)_ × N_eDNA(free);_ *N*_eDNA(network)_ ≡ no. of network-bound eDNA in chosen compartment; *N*_eDNA(free)_ ≡ no. of free eDNA in an adjacent compartment]	See main text, rule ii, in the section describing construction of the agent- based *in silico* model
7	Tfp-driven NTHI movement on eDNA network	NTHI in a compartment moves to an adjacent compartment when both compartments belong to the eDNA network	*k*_Tfpdna_ = 3.12 μm/min (*k*_7_ = *k*_Tfpdna_ × *N*_NTHI_)	In biofilms formed by *P. aeruginosa*, the bacteria move on eDNA tracks via Tfp movements; avg displacement ≈5 μm/100 s (3.12 μm/min) (20). It is currently unknown whether NTHI moves on eDNA strands using Tfp.
8	Replication of planktonic NTHI	N_pNTHI_ and *N*_c_ in a compartment increases and decreases by 1, respectively (*N*_pNTHI_ → N_pNTHI_ + 1; *N*_c_ → *N*_c_ – 1)	pNTHi replication rate (*k*_repli_) = 0.0167 NTHI particles/min (*k*_8_ = *k*_repli_ × *N*_pNTHI_)	Same as rule 1
9	eDNA production by planktonic NTHI	Same as rule 2	Same as rule 2	Same as rule 2
10	Nutrient addition	At intervals of 16 h and 8 h, nutrient densities in all compartments are reset to 2 (*N*_c_ → *N*_c_ = 2)	NA^a^	This rule represents change of medium in biofilm static culture every 16 h and 8 h
11	NTHI removal from supernatant	At intervals of 16 h and 8 h, planktonic NTHI in each compartment is removed (probability of 0.8)	NA	This rule represents removal of NTHI in supernatant when medium in biofilm static culture is replaced every 16 h and 8 h
A^b^	Mass movement of NTHI	Excess NTHI cells above *n*_thres_ (*N*_NTHI_ − *n*_thres_) in compartment are transferred to adjacent compartment with room for that amount; transfer is done at every MC step at time interval Δ*t* of 0.1 min		Transfer of excess NTHI to neighboring compartments represents mass movement of NTHI due to physical forces between bacteria due to tight packing of finite- sized NTHI particles; similar movements have been considered in other *in* *silico* biofilm models (50)
B^b^	Nutrient diffusion	Nutrient concentration is homogenized at every MC step at time interval Δ*t* of 0.1 min	We assumed nutrient diffusion (*D*) of 100–1,000 μm^2^/s	Nutrient variable in model represents range of molecule sizes, from small metabolites to large proteins; nutrient particles travel rate (*D*Δ*t*)^½^ of ≈24– 77 μm in Δ*t*, so homogenization of nutrients in simulation box (128 μm × 40 μm) is reasonable

^a^ NA, not applicable.

^b^ Rules A and B are implemented at every MC step.

The NTHI bacterial cells were inoculated at time *t* = 0 into the compartments along the *z* = 0 line (substrate) with a low concentration of bacterial cells per compartment (=0.2 cells/μm^3^). We simulated the biofilm formation until 88 h. The NTHI cells inoculated into the compartments replicated and moved to neighboring compartments once the number of NTHI cells in a compartment increased beyond the threshold number. The NTHI cells also spread due to their movements along the eDNA network. This resulted in spatial growth of NTHI cells on the substrate (Fig. 4B to D). eDNA strands were produced by a small subset of NTHI cells. These eDNA strands diffused into the neighboring compartments or became connected to the eDNA network (Fig. S2). The profiles of the biofilms generated in the *in silico* model were qualitatively similar to their *in vitro* counterparts (Fig. 4E to G) in the following ways. Similar to the biofilms formed *in vitro*, the NTHI towers in the biofilm formed *in silico* grew in height and width and at later times (*t* ≥ 40 h) the growing NTHI clusters merged with each other (Fig. 4B to G) to generate smoother biofilm surfaces. In addition, at later times (≥40 h), uneven structures at short length scales (≤5 μm) occurred in the biofilms formed *in vitro* and *in silico*, where regions with low and high NTHI bacterial cell numbers coexisted with each other (Fig. 4B to G). These similarities are further quantified in the section below. The dispersion of the NTHI cells to the planktonic state resulted in removal of NTHI from the system during the feeding times. The slower dispersion of the bacteria from the compartments containing the eDNA network generated a spatially uneven organization of NTHI within the biofilm (Fig. 4B to D). Each model simulation produced a microscopically different biofilm configuration due to the statistical differences in the initial inoculation configurations and the stochastic nature of the dynamic processes (e.g., NTHI replication, eDNA production) involved in the biofilm growth. Next, we quantitatively compared the biofilm structures in the simulation with those formed in the *in vitro* culture.

10.1128/mBio.01466-17.3 FIG S2  Kinetics of an eDNA network for biofilms formed *in silico*. The figure shows a representative configuration of the eDNA network in the *in silico* model at *t* = 16 h (A), *t* = 40 h (B), and *t* = 88 h (C). Download FIG S2, PDF file, 0.2 MB. Copyright © 2017 Das et al. 2017 Das et al. This content is distributed under the terms of the Creative Commons Attribution 4.0 International license.

We validated the model simulations against the biofilm experiments by comparing, (i) the kinetics of the average biofilm height (*h*_avg_), (ii) the kinetics of the coefficient of variation of the biofilm profile, (iii) the variation of *C*_z*_(*r*,*t*) with *r* at a fixed *z* plane (*z* = z*) at different times (*t*) of biofilm growth, and (iv) variation of *C*_*z*_(*r*,t*) with *r* at multiple *z* planes for a fixed *t* (=t*). We focused on qualitatively comparing the morphological patterns between the biofilms formed *in silico* and *in vitro*, e.g., whether surface fractal structures were present or whether the θ values were less than unity in both these biofilms. This was more appropriate than a quantitative comparison in our case, because a quantitative comparison of the spatial patterns would compare both biofilms in terms of their associations with a specific type of a surface fractal or with a particular value of θ. Since the *in silico* model provides a reduced or coarse-grained description of the biofilms formed *in vitro*, many microscopic details responsible for generating the precise nature of the surface fractals are not explicitly present in the *in silico* model, and thus the *in silico* biofilm morphologies are unsuitable for quantitative comparisons.

**FIG 5:**
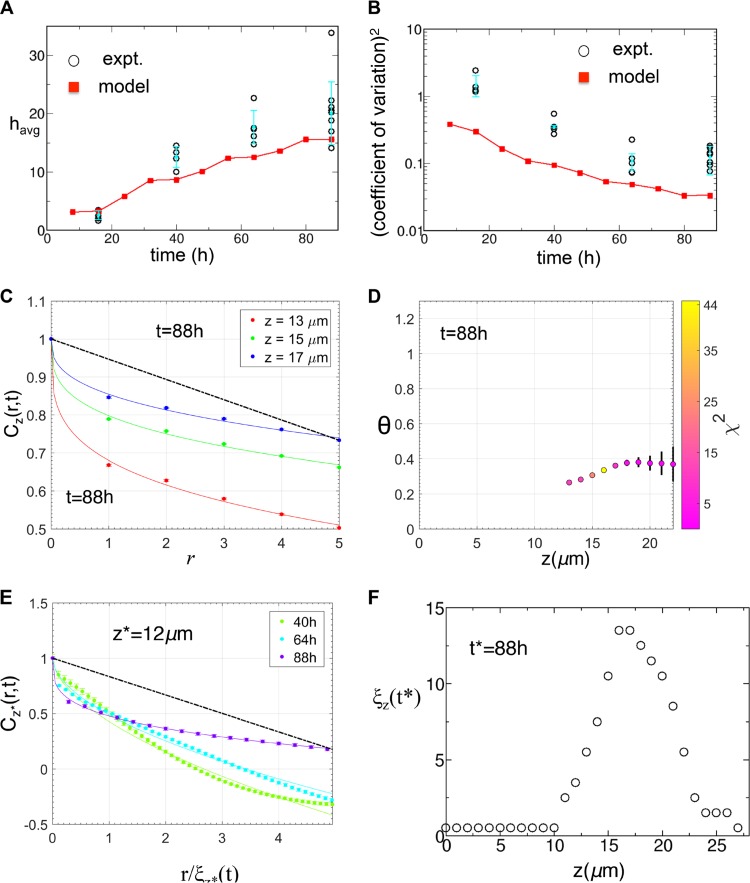
Comparison of the biofilm profiles between the *in silico* model and the *in vitro* experiments for WT NTHI. (A) The kinetics of the average biofilm height (*h*_avg_) obtained from the *in silico* model (red squares) was compared against that for the *in vitro* NTHI biofilms (circles). The average heights for the *in vitro* NTHI biofilms were calculated at *t* = 16 h, 40 h, 64 h, and 88 h for multiple replicates. *h*_avg_ for each replicate is displayed. The mean values of *h*_avg_ calculated for different biofilm replicates formed *in vitro* and the associated error bars are shown in filled circles in cyan. (B) Comparison of the (coefficient of variation)^2^, calculated for the biofilm surfaces obtained from *in silico* simulations (red squares) and *in vitro* experiments (circles). The mean values of the (coefficient of variation)^2^ calculated for different biofilm replicates formed *in vitro* and the associated error bars are shown in filled circles in cyan. (C) Variation of *C*_*z*_(*r*,t*)/C_*z*_(0,t*) with *r* at short distances (≤5 μm) for multiple *z* planes for the *in silico* biofilm at t* = 88 h. The data were averaged over 30,000 initial configurations. The solid lines show the fits of equation 3 to the data. All the fits produces θ values of <1, in qualitative agreement with the *in vitro* data (Fig. 2A and B). The dashed black line shows the θ = 1 case as a reference. (D) Values of θ at different *z* planes obtained by fitting the *C*_*z*_(*r*,t*)/*C*_*z*_(0,t*) data for the *in silico* biofilm at t* = 88 h. θ values are not shown where the *C*(*r*,*t*) decreased substantially in length scales of *r* ≤ 1 μm, comparable to the compartment size (*l*_0_ = 1 μm). The θ values close to the biofilm surface also are not shown, due to large errors in the estimations due to the large errors in the data for *C*_*z*_(*r*,t*) near the biofilm surfaces. The symbol colors display the χ^2^ values for the fits used to estimate the θ values. (E) Variation of *C*_z*_(*r*,*t*)/*C*_*z*_(0,t*) with *r*/ξ_z*_(*t*) at larger distances for multiple times (t* = 40 h, 64 h, and 88 h) for the *in silico* biofilm at z* = 12 μm. The data show any absence of scaling, which is in agreement with the *in vitro* data (Fig. 3A). (F) Variation of the correlation length ξ_*z*_(t*) (unit, μm) with *z* at t* = 88 h. The initial rise in ξ_*z*_(t*) followed by a decrease as we approached the biofilm tip from the substrate was in qualitative agreement with the *in vitro* data (Fig. 3B, inset).

We simulated NTHI growth for 88 h and calculated the above quantities based on the *in silico* morphology of the NTHI biofilm at regular time intervals. The above quantities (e.g., *h*_avg_) were averaged over at least 1,000 *in silico* biofilm configurations obtained from independent simulation runs that were initiated with the same model parameters but with different random realizations of the initial NTHI inoculation. The biofilm height in the model was determined by finding a biofilm surface (the number of NTHI bacterial cells in a compartment that crossed a specific threshold value the first time) as the biofilm was approached from the top along the *z* axis (see Materials and Methods). The two-dimensional biofilm surface in confocal images of the biofilms was found similarly using a threshold in the image intensity. The coefficients of variation (CVs) for the biofilm surfaces were calculated using following definition, CV = σ/*h*_avg_, where σ is the standard deviation for the fluctuations in the biofilm surface height across the average value *h*_avg_ (see Materials and Methods and Text S1). The coefficient of variation is a dimensionless parameter that quantifies the magnitude of fluctuations in the biofilm surface height relative to the average height. A larger coefficient of variation is indicative of larger fluctuations in the biofilm height across the average value. The average biofilm height increased, but the rate of its increase slowed with time in the model (Fig. 5A). This behavior was in qualitative agreement with the results of the *in vitro* experiments (Fig. 5A). The coefficient of variation for the biofilm profile decreased with time, as the rate of decrease slowed down at later times (Fig. 5B). This behavior was also in qualitative agreement with results in the *in vitro* experiments (Fig. 5B).

Next we calculated *C*_*z*_(*r*,*t*) for the NTHI morphologies at different time points (16, 40, 64, and 88 h after the initial inoculation). Similar to the *in vitro* morphologies, *C*_*z*_(*r*,*t*) at low values (≤5 μm) of *r*, decreased as 1 − *ar*^θ^, where θ is <1 (Fig. 5C and D) for several *z* planes for biofilm growth. This demonstrated the presence of fractal structures in the interface between the NTHI-rich and -poor regions in the *in silico* model. We also calculated the correlation length ξ_*z*_(t*) at a *z* plane by using *C*_*z*_(*r*,t*) for NTHI morphologies at a fixed time, t*. *C*_*z*_(*r*,t*) failed to scale with *r*/ξ_*z*_(t*) at different *z* planes at a fixed *t* (=t*) (Fig. S3), or *C*_z*_(*r*,*t*) failed to scale with *r*/ξ_z*_(*t*) at different values for different values of *t* at a fixed *z* plane (*z* = z*) (Fig. 5E). This behavior was also in qualitative agreement with the NTHI profiles for biofilms formed *in vitro*. Similar to the morphologies formed *in vitro* (Fig. 3B, inset), ξ_*z*_(t*) at a fixed *t* (=t*) first increased with *z* and then decreased as *z* approached the tip of the biofilm (Fig. 5F). There were few qualitative disagreements between the model and *in vitro* data; some of the disagreements arose due to the finite system size effects in the model. The early biofilm growth (16 h) in the model showed θ of <1 (Fig. S4), whereas the biofilm morphologies formed *in vitro* at a few *z*-planes generated θ values of <1 at *t* = 16 h. This discrepancy could have arisen due to later (>16 h) induction of bacterial dispersion in the biofilms formed *in vitro*. Also, *C*_*z*_(*r*,*t*) becomes negative at larger values of *r* (>ξ_*z*_(*t*)) in the model, whereas *C*_*z*_(*r*,*t*) when calculated from the confocal imaging data for the same values of *r*/ξ_*z*_(*t*) remains positive. This discrepancy between the model and experiments arises due to the influence of the finite size of the lateral (*x-y*) dimensions for the biofilms formed *in vitro* and *in silico*. The confocal images showed the presence of larger structures of sizes >40 μm, comparable to the size (142 μm by 142 μm) of the biofilm area studied in the microscopy experiments. Therefore, the calculation of *C*_*z*_(*r*,*t*) at these larger length scales was influenced by effects due to finite sample sizes, e.g., incomplete statistical representation of spatial structures that were on the order of or larger than the biofilm sample. Resolution of this issue will require imaging of larger areas in the biofilm. The finite size of the system also affected the calculation of *C*_*z*_(*r*,*t*) in the *in silico* model at these length scales, which could be a possible reason behind the disagreement between the model and the data at larger length scales. Furthermore, many molecules, such as polysaccharides, that were not considered in the *in silico* model are likely to be present in the NTHI biofilms formed *in vitro* as a part of the EPS. The EPS can slow nutrient diffusion in *in vitro* culture (28) to produce nutrient gradients at larger length scales (≥20 μm). These effects could be responsible for the differences found between the biofilms formed *in vitro* and *in silico* at these length scales, e.g., with spikier patterns in biofilms formed *in vitro* compared to those formed *in silico* (Fig. 4B and C).

10.1128/mBio.01466-17.4 FIG S3  Absence of scaling with a single characteristic length scale, ξ_z_(t*), in biofilms formed *in silico* at 88 h. The figure shows the variation of C_z_(*r*,t*)/C_z_(0,t*) with *r*/ξ_z_(t*) at larger distances for multiple *z* planes (14 μm, 16 μm, and 19 μm) for the *in silico* biofilm at t* = 88 h. The data show any absence of scaling, which is in agreement with the *in vitro* data (Fig. 3B). Download FIG S3, PDF file, 0.3 MB. Copyright © 2017 Das et al. 2017 Das et al. This content is distributed under the terms of the Creative Commons Attribution 4.0 International license.

10.1128/mBio.01466-17.5 FIG S4  Analysis of the pair correlation in biofilms formed *in silico* at 16 h. (A) The figure shows the variation of C_z_(*r*,t*)/C_z_(0,t*) with *r* at short distances (< 5 μm) for multiple *z* planes with the *in silico* biofilm at t* = 16 h. The data were averaged over 1,000 initial configurations. The solid lines show the fits of equation 2 to the data. All the fits produced θ values less than unity. (B) Variation of θ with the *z* planes for the data shown in panel A. Download FIG S4, PDF file, 0.5 MB. Copyright © 2017 Das et al. 2017 Das et al. This content is distributed under the terms of the Creative Commons Attribution 4.0 International license.

### Fractal structures in *in vitro* wild-type NTHI biofilms arise due to the presence of eDNA, as shown via model predictions with the Δ*comE* mutant strain of NTHI.

**FIG 6:**
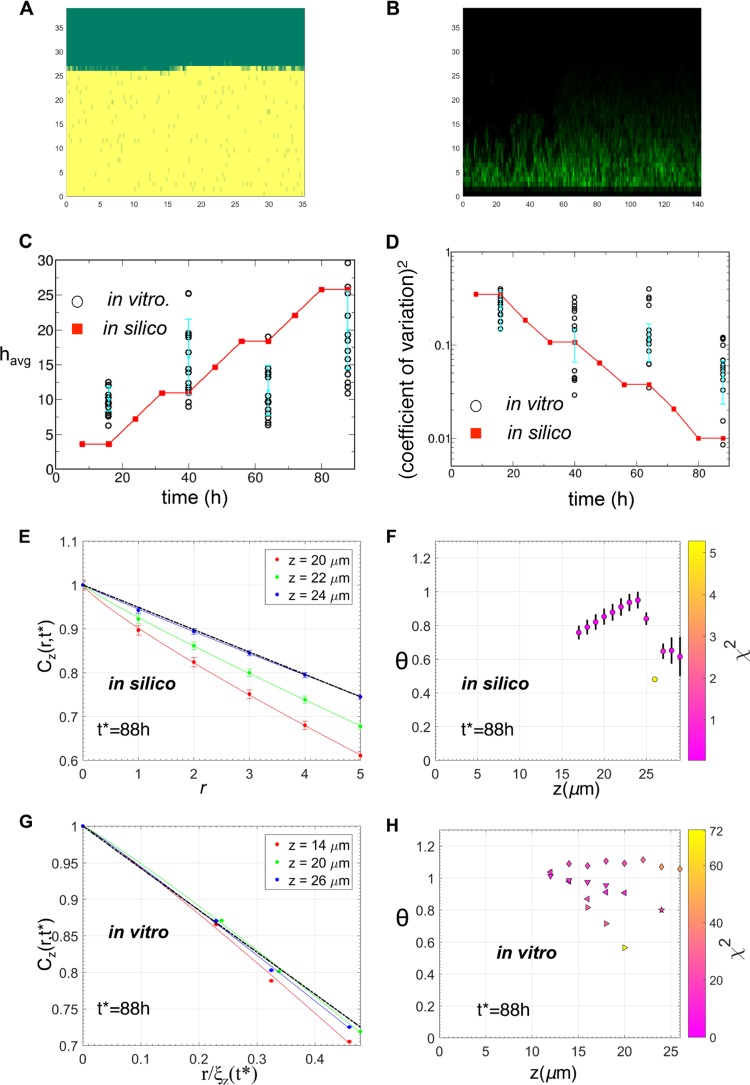
Test of model predictions with biofilms formed *in vitro* by the Δ*comE* mutant strain. (A) A configuration of the mutant Δ*comE* NTHI cells at *t* = 88 h in the *in silico* model. (B) Organization of the mutant Δ*comE* NTHI cells in the confocal image for a Δ*comE* strain biofilm formed *in vitro* at *t* = 88 h. (C) Kinetics of the average biofilm height (*h*_avg_) obtained from the *in silico* model (red squares), with the NTHI Δ*comE* mutant compared against the *in vitro* Δ*comE* strain biofilms (circles). The average heights for the mutant Δ*comE* biofilms were calculated at *t* = 16 h, 40 h, 64 h, and 88 h for multiple replicates. *h*_avg_ for each replicate is displayed. The mean values of *h*_avg_ calculated for different biofilm replicates formed *in vitro* and the associated error bars are shown as filled circles in cyan. (D) Comparison of the (coefficient of variation)^2^ calculated for the biofilm surfaces obtained from *in silico* simulations (red squares) and *in vitro* experiments with the mutant Δ*comE* (circles). The mean values of the (coefficient of variation)^2^ calculated for different biofilm replicates formed *in vitro* and the associated error bars are shown in filled circles in cyan. (E) Variation of *C*_*z*_(*r*,t*)/*C*_*z*_(0,t*) with *r* at short distances (≤5 μm) for multiple *z* planes for the *in silico* biofilm at t* = 88 h. The data were averaged over 30,000 initial configurations. The solid lines show the fits of equation 3 to the data. All the fits produced θ values of ≈1. The dashed black line shows the θ = 1 case as a reference. θ values are not shown where the *C*(*r*,*t*) decreased substantially in length scales (*r* ≤ 1 μm), comparable to the compartment size (*l*_0_ = 1 μm). The θ values close to the biofilm surface also are not shown due to large errors in the estimation of *C*_*z*_(*r*,t*). (F) Values of θ at different *z* planes obtained by fitting the *C*_*z*_(*r*,t*)/*C*_*z*_(0,t*t) data for the *in silico* biofilm at t* = 88 h. The χ^2^ values, indicating the quality of the fits used to estimate the θ values, are shown by the color of the symbols. (G) Variation of *C*_*z*_(*r*,t*)/*C*_*z*_(0,t*) with *r*/ξ_*z*_(t*) at short distances (≤5 μm) for multiple *z* planes for the mutant Δ*comE* biofilm at t* = 88 h. Values of θ at different *z* planes were obtained by fitting the *C*_*z*_(*r*,t*)/C_z_(0,t*) data. The estimated θ values were in qualitative agreement with that for the model predictions in panels E and F. (H) Values of θ at different *z* planes obtained by fitting the *C*_*z*_(*r*,t*)/*C*_*z*_(0,t*) data for the mutant Δ*comE* biofilm at t* = 88 h. For the majority of the planes, θ was close to 1, as predicted by the *in silico* model (panel F). The χ^2^ values indicating the quality of the fits used to estimate the θ values are shown by the colors of the symbols.

We used the *in silico* model to determine mechanisms underlying the fractal structures present at short length scales (≤5 μm) in the biofilm morphologies. We found that in simulations where the eDNA network was limited and NTHI cells dispersed at a very low rate (~100 times lower than the WT) in all compartments, then the fractal nature of the interfaces in the biofilms was mostly absent. The absence of these fractal structures generated the short distance (≤5 μm) decay of *C*_*z*_(*r*,*t*) as 1 − *ar*^θ^, where the θ value is ≈1. In addition, the simulations under the above condition produced smoother biofilm surfaces (Fig. 6A) with smaller coefficients of variation than with the biofilms formed *in silico* by the wild-type NTHI. We compared the above predictions to experimentally generated values obtained from biofilms formed by NTHI strain Δ*comE*, which potentially mimicked the above *in silico* conditions. Because the ComE pore is required for DNA release into the environment, biofilms formed by strain Δ*comE* (Fig. 6B) contain very little eDNA compared to the parent strain (19). In addition, the ComE pore is also required for Tfp expression through the NTHI outer membrane. Thus, the Δ*comE* mutant also lacks a fully functional Tfp, which could potentially give rise to a lower rate of dispersion than the WT strain. Biofilms formed *in vitro* were generated using the same protocol for biofilm seeding and maintenance as used for the wild-type NTHI strain. The predictions regarding the kinetics of the average height and the coefficients of variation for the biofilms generated using the *in silico* model for Δ*comE* strain bacteria produced minimal amounts of eDNA, which was dispersed into the surrounding liquid medium at a lower rate and did not generate any twitching motility on eDNA strands in all the compartments, in qualitative agreement with the corresponding results for the *in vitro* Δ*comE* mutant strain biofilms (Fig. 6C). In both the *in silico* model and the *in vitro* experiments, the older (e.g., 88 h) strain Δ*comE* biofilms produced smoother biofilm surfaces (or lower coefficients of variation) (Fig. 6D). The calculation of *C*_*z*_(*r*,t*) for the NTHI densities for 88 h biofilms generated by the *in* silico simulations with the mutant NTHI strain generated bacterial clusters with smoother but sharper interfaces (Fig. 6E and F). This was reflected in the values for the θ exponent, which for the majority of the *z* planes was close to 1 (Fig. 6F). This prediction agreed with the behavior of *C*_*z*_(*r*,t*) calculated for the strain Δ*comE* biofilms formed *in vitro* at t* = 88 h (Fig. 6G and H). In the *in silico* simulations for the Δ*comE* mutant strain, the lower rate of dispersion of bacteria as well as a substantial decrease of the motility of the bacterial cells along the eDNA network resulted in more homogeneous distributions of bacterial cells within the biofilms and smoother biofilm surfaces than with the wild-type NTHI biofilms formed *in silico*.

## DISCUSSION

We quantitatively characterized the spatial organization of bacterial cells within NTHI biofilms formed *in vitro* by analyzing pair correlations between bacterial densities in confocal images of the biofilms. Our analysis revealed the presence of scale invariant or fractal structures at the interfaces separating the wild-type NTHI bacterial cells from the surrounding liquid. These interfaces occurred in shorter length scales (≤10 μm) (Fig. 1E; Fig. S1). The presence of the fractal patterns can have several functional implications. The fractal interfaces increase the surface area of the NTHI biofilm, thereby exposing NTHI cells to the surrounding liquid, and this can facilitate greater absorption of nutrients from the environment. This could be particularly important for NTHI survival in the nutrient-poor environment of the middle ear. Furthermore, theoretical models investigating diffusion of particles on fractal landscapes (29, 30) have shown the possibility of trapping the diffusing particles in a spatial location, because a fractal landscape can produce tortuous open spaces that can trap diffusing particles, like an “ant in the labyrinth” (31). Thus, the fractal interfaces in NTHI biofilms could trap molecules such as autoinducer-2 (AI-2), which is secreted by NTHI bacteria in a spatial location, and help mediate quorum signaling in that region. The localization of the AI-2 molecules could potentially generate spatial feedback for quorum sensing. However, the presence of the fractal surfaces also produces more regions with low bacterial cell densities, which could in turn lead to lower production of the quorum-sensing molecules. Thus, a trade-off between the above-described effects will determine how the fractal structures in a NTHI biofilm affect quorum sensing. While the effects described above can help in the survival of NTHI, the fractal structures can also increase the exposure of the biofilms to antimicrobial proteins, phagocytic cells, and other host immune effectors. Therefore, the beneficial or detrimental role of the presence of the fractal structures depends on how these opposing effects are balanced in a host environment. Our analysis also revealed the presence of multiple characteristic length and time scales in the biofilm morphologies (Fig. 3), implying the relevance of multiple dynamic processes (e.g., NTHI cell growth, NTHI cell mass movements and dispersion, formation of eDNA network) that span a range of length and time scales in regulating NTHI biofilm morphology. Therefore, altering specific features (e.g., changes in the fractal patterns) of NTHI biofilm architecture will likely require perturbing several processes simultaneously.

How unique are the fractal structures formed by NTHI biofilms? The existence of fractal structures within bacterial biofilms has been reported for a few biofilm morphologies, e.g., biofilms formed *in vitro* by activated sludge (32). The majority of the analyses of confocal images of bacterial biofilms in the recent literature were performed with the software package COMSTAT2 (33) and characterized the biofilms in terms of biomass, average height, and surface roughness. Thus, it is unclear whether fractal structures at short length scales are present in these studies. To address this question, we analyzed biofilms formed by another major OM pathogen, *Moraxella catarrhalis*, using *C*_*z*_(*r*,*t*) values calculated at multiple *z* planes. We found that for smaller length scales (<1.5 μm), the exponent θ is close to unity for most of the *z* planes, which indicated a sharp nonfractal interface (Fig. 1D) between the *M. catarrhalis* cells and the surrounding medium (Fig. S5). Thus, fractal patterns at short length scales along the biofilm-liquid interface are not universal, and understanding how these structures arise in biofilms formed by different bacterial species under different experimental conditions is an interesting future direction of research.

10.1128/mBio.01466-17.6 FIG S5  Analysis of biofilm morphologies formed by *M. catarrhalis*. (A) Variation of *C*_z_(*r*,t*)/*C*_*z*_(*r* = 0,t*) with *r* at a fixed t* (=88 h) and with different *z* planes within a replicate of the *in vitro* biofilm (blue points). The corresponding data for the NTHI (red points) biofilm at 88 h are shown as a reference. The θ exponent was obtained by fitting the function in equation 3 for the data on multiple *z* planes. The fits are shown with solid lines. The *M. catarrhalis* data can be fit well with a straight line (cyan line) that corresponds to θ = 1. (B) Values of the estimated θ exponent for different *z* stacks calculated by fitting equation 2 to *C*_*z*_(*r*,t*) for an *r* value of ≤1.5 μm, calculated for 7 different replicates for the 88 h *M*. *catarrhalis* biofilm. The symbol colors show the χ^2^ values for the fits that were used to estimate the θ values. The smaller the χ^2^ value, the better the fit. Download FIG S5, PDF file, 0.6 MB. Copyright © 2017 Das et al. 2017 Das et al. This content is distributed under the terms of the Creative Commons Attribution 4.0 International license.

We developed an *in silico* agent-based model to investigate the mechanistic roles of NTHI replication, eDNA, and Tfp expression in giving rise to fractal structures in NTHI biofilms. Recent studies have identified several candidates, including eDNA (8, 9, 11), quorum signals (7), and Tfp (13, 14, 34), as important regulators of the morphology of NTHI biofilms formed *in vitro*. The processes considered in the agent-based model were based on the known information about NTHI biofilm biology. We studied the roles of eDNA and Tfp in influencing the growth and structure of NTHI biofilms by explicitly considering potential eDNA-NTHI interactions in the *in silico* model. We hypothesized two different types of interactions between NTHI cells and the eDNA. (i) NTHI adheres to and ratchets along eDNA strands via twitching motility generated by Tfp. (ii) NTHI dispersion from the biofilm to surrounding liquid, induced by Tfp and quorum signals, occurs at a lower rate in the presence of the eDNA network. The growth and structure of the *in silico* biofilms agreed qualitatively with that in the NTHI biofilms formed *in vitro*. The role of the above interactions between the NTHI and eDNA in giving rise to the fractal patterns in wild-type NTHI biofilms was investigated further by analyzing spatial patterns in *in silico* biofilms for an NTHI mutant that produced substantially less eDNA within the biofilm matrix and showed a low dispersion rate and impaired motility along the eDNA network. The biofilm morphologies for the *in silico* mutant were compared with that of biofilms formed *in vitro* by a Δ*comE* mutant of NTHI. The biofilms formed by the Δ*comE* mutant contained substantially less eDNA than the wild type, and the Tfp function was impaired in the Δ*comE* strain. As predicted by the *in silico* simulation, the *ΔcomE* strain biofilms displayed a considerable loss of the fractal patterns found in the wild-type biofilms and smoother biofilm surfaces (Fig. 6). The biofilms formed by the *pilA* mutant of NTHI, in which NTHI cells are devoid of Tfp function and thus are unlikely to possess the NTHI-eDNA interactions hypothesized above, also showed similar loss of the fractal patterns in the biofilm morphologies (Fig. S6). These findings support the hypothesized interactions between NTHI and eDNA in biofilms.

10.1128/mBio.01466-17.7 FIG S6  Loss of fractal interfaces in the *in vitro* biofilms formed by the *pilA* mutant of NTHI. (A) Variation of *C*_*z*_(*r*,t*)/*C*_*z*_(0,t*) with *r* at short distances [*r*/ξ_*z*_(t*) ≤ 1] for multiple *z* planes for the *in vitro* biofilm formed by the *pilA* mutant of NTHI at t* = 88 h. The biofilm was grown using the same protocol as for wild-type NTHI (see Materials and Methods). The solid lines show the fits of equation 3 to the data. All the fits produced θ values (0.74, 0.73, 0.66) closer to unity than that of WT NTHI (Fig. 2). This indicates a loss of fractal interfaces in the biofilms formed by the *pilA* mutant compared to its wild-type counterpart. The dashed black line shows the θ = 1 case as a reference. θ values are not shown where the *C*(*r*,*t*) values decreased substantially (more than half of *C*_*z*(0,*t*)_) for *r* ≤ 1 μm. (B) Values of θ on different *z* planes obtained by fitting *C*_*z*_(*r*,*t*)/*C*_*z*_(0,*t*) versus *r* data for the *in vitro* biofilms formed by the *pilA* mutant at t* = 88 h. The θ values are shown for two replicates. The χ^2^ values indicate the quality of the fits used to estimate the θ values and are shown by the colors of the symbols. Download FIG S6, PDF file, 0.5 MB. Copyright © 2017 Das et al. 2017 Das et al. This content is distributed under the terms of the Creative Commons Attribution 4.0 International license.

We used an agent-based model to describe the kinetics of NTHI biofilm formation. To the best of our knowledge, this is the first *in silico* model that describes the formation of NTHI biofilms. The agent-based model described the biofilm formation in terms of agents, such as NTHI bacterial cells, eDNA strands, and nutrients, that interact with each other with specific rules representing relevant biological processes, e.g., NTHI replication. An advantage of agent-based modeling is the flexibility to introduce rules that can be used as hypotheses to probe specific interactions between the components (e.g., NTHI bacteria and eDNA) of interest in a biological system. Agent-based models have been widely used in modeling generic biofilm growth (24, 35–37) as well as in a wide range of host-pathogen systems (38) where spatial heterogeneity plays an important role. However, these biofilm models did not explicitly investigate the role of eDNA in regulating biofilm morphologies. Biofilm models based on other methods, such as partial differential equations (39–41) and Brownian dynamics (42), have also been used previously to describe generic biofilm formation or biofilm formation in specific bacterial species, such as *P. aeruginosa* (41) and *Bacillus subtilis* (40, 43). These models usually entail microscopic details pertaining to specific bacterial species or contain simplifying assumptions; therefore, they cannot be readily used for describing NTHI biofilm formation.

How accurate is the *in silico* model in describing biofilm formation by other bacterial species? The agent-based model includes distinct hypotheses pertaining to NTHI biofilm formation, such as Tfp-induced movements on the eDNA network and the influence of Tfp and eDNA on NTHI dispersion. Though eDNA is found in the EPS of biofilms formed by many other bacterial species, such as *M. catarrhalis*, and many other bacterial species (e.g., *P. aeruginosa*) express Tfp, it is unclear if the specific hypotheses regarding the interactions between eDNA and Tfp in NTHI biofilms can be generalized to other bacterial species. Therefore, based on this study’s findings, we anticipate that the *in silico* model can be used to analyze biofilms formed by the wild-type or mutant NTHI strains but the model as it stands cannot be readily applied to describe biofilm formation in another bacterial species, such as *M. catarrhalis*, *S. aureus*, or *P. aeruginosa*. However, the *in silico* model still contains the flexibility of an agent-based modeling framework (38), where the existing rules in a model can be modified and new rules can be added easily; therefore, the current model can be extended to describe biofilm formation by other species.

What do the results obtained here mean for biofilms formed *in vivo*? Since the middle ear presents NTHI with a nutrient-poor and hostile environment, we expect that the survival of NTHI would require formation of these fractal structures. The *in silico* analysis and the agent-based model can be easily extended to analyze NTHI biofilm formation *in vivo*. The irregular three-dimensional shape and larger size (~100 mm) (44) of the middle ear cavity in *Chinchilla lanigera*, an animal model for experimental OM, will likely play an important role in determining formation and kinetics of NTHI biofilms *in vivo*. Therefore, an extension of the current *in silico* model will require simulations of biofilm growth in three dimensions in larger (>10 times) system sizes and should also incorporate explicit nutrient diffusion, as the current model assumption of instantaneous equilibration of the nutrient density will not be appropriate at larger distances. Another useful improvement for the *in silico* model will be to include a more detailed model for eDNA network formation based on microscopic details of the eDNA network informed by *in vivo* imaging experiments (11). Such an effort is likely to offer exciting mechanistic insights regarding the role of the eDNA network and Tfp expression in regulating NTHI biofilm structures in the middle ear.

## MATERIALS AND METHODS

### Analysis of *in vitro* NTHI biofilm morphology based on the pair correlation function.

We calculated the pair correlation function between the bacterial densities at two locations, ${\overset{\to }{r}}_{1}\equiv (x,\;y,\;z)$ and ${\overset{\to }{r}}_{2}\equiv (x+r_{x},\;y+r_{y},\;z)$ in a plane (*x-y*) parallel to and residing at a fixed distance (*z*) from the substrate (Fig. 1A). The pair correlation function *C*_*z*_(*r*,*t*) is widely used in statistical physics (22, 23, 26) and materials science (21) for characterizing spatial structures. *C*_*z*_(*r*,*t*) is defined as shown in equation 1:

(1) $$C_{z}(r,t)=〈1/L^{2}\underset{x,y}{\sum }\rho (x,y,t)\rho (x+r_{x},y+r_{y},z,t〉=〈{\overset{¯}{C}}_{z}(r,t)〉$$

where $r=\left|{\overset{\to }{r}}_{1}-{\overset{\to }{r}}_{2}\right|=\sqrt{\left(r_{x}{}^{2}+r_{y}{}^{2}\right)}$ denotes the distance between the two locations in the *x-y* plane and *L* is the length of the sample along the *x* or *y* direction. In the above expression, it has been assumed that the bacterial density in the *x-y* plane depends on the magnitude of the relative distance between the vectors ${\overset{\to }{r}}_{1}$ and ${\overset{\to }{r}}_{2}$ (translational symmetry) and does not depend on the direction of the vector $\left({\overset{\to }{r}}_{1}-{\overset{\to }{r}}_{2}\right)$(rotational invariance). The angular brackets indicate an average over an ensemble of density configurations. The behavior of *C_z_*(*r*,*t*) at short distances or small values of *r* can be used to determine the nature of the spatial patterns within that length scale. When *r* is larger than a microscopic length scale *a*_0_ (e.g., spatial resolution of the confocal images or length scales ≈0.27 μm) but smaller than an intermediate scale *w*, i.e., $a_{0}\leq r\leq w$, *C_z_*(*r*,*t*) can be approximated by using equation 2 (21, 22):

(2) $$C_{z}(r,t)/C_{z}(r=0,t)\approx 1-ar^{\theta }$$

The value of the exponent *θ* contains information regarding the nature of the spatial pattern in that length scale (21–23, 26). For example, if the bacterial cluster, embedded in a space of dimension *d*, forms a scale-invariant structure or a mass fractal (Fig. 1B) of dimension *d*_m_ (Hausdorff dimension) (0 < *d*_m_ < *d*), then θ = *d*_m_ − *d*. When the bacterial cluster forms a regular solid structure associated with a fractal (Fig. 1C) or a sharp (Fig. 1D) interface separating regions rich and poor in bacterial density, then the equation θ = *d* − *d*_s_, and θ = *d* − (*d* − 1) (Porod’s law [22, 27]), respectively. *d*_s_ (*d* > *d*_s_ > *d −* 1) denotes the dimension of the fractal surface. In our analysis, *d* = 2, therefore, *θ* of <1 will indicate the presence of bacterial density in a surface fractal pattern. If *θ* = 1, it will indicate the presence of a sharp but nonfractal interface, and a θ value of <0 will indicate the presence of bacterial clusters with mass fractal structures. We found that the *in vitro* NTHI biofilm morphologies produced different types of surface fractals at different length scales that were characterized by different values of the θ exponent, i.e., the surface fractal crosses over from one type to another type as the distance *r* increases beyond the length scale *r*_*c*_ (see Fig. S1) (22). This behavior can be described in terms of *C*_*z*_(*r*,*t*) as shown in equation 3:

(3) $$C_{z}(r,t)/C_{z}(r=0,t)\approx 1-a_{<}r^{\theta^{<}}$$

when *r* < *r_c_*, and when *r* > *r_c_*, equation 3 is as follows:

$$C_{z}(r,t)/C_{z}(r=0,t)\approx 1-a_{>}r^{\theta >}$$

where $r_{c}={\left(\frac{a_{>}}{a_{<}}\right)}^{\theta_{<}-\theta_{>}}$. The changes in equation 3 are well described by a crossover function (22) (Fig. S1), as shown in equation 4:

(4) $$C_{z}(r,t)/C_{z}(r=0,t)\approx 1-\left[1-g(r,r_{c})\right]a_{<}r^{\theta_{<}}-g(r,r_{c})a_{>}r^{\theta_{>}}$$

where *g*(*r*,*r*_*c*_) = *r*^*m*^/(*r*^*m*^ + *r*_*c*_^*m*^) and *m* = 5.

We also attempted to fit *C*_*z*_(*r*,t*) for t* = 88 h for a larger range of *r* values (0 to 69.6 μm) via a function described by a combination of different power law functions in three segments: 0 ≤ *r* ≤ *r*_1_, *r*_1_ < *r* ≤ *r*_2_, and *r*_2_ < *r* ≤ 69.6 μm. The fitting function was constructed by considering crossover behavior similar to that in equation 4, resulting in equation 5:

(5) $$C_{z}(r,t)/C_{z}(r=0,t)\approx 1-\left[1-g(r,r_{1})\right]a_{1}r^{\theta_{1}}-\left[g(r,r_{1})-g(r,r_{2})\right]a_{2}r^{\theta_{2}}=g(r,r_{2})a_{3}r^{\theta_{3}}$$

where *g*(*r*,*r*_1_) = *r*^*m*^/(*r*^*m*^ + *r*_*i*_^*m*^), *i* = {1,2}, *r*_1_ > *r*_2_, and *m* = 5.

Equation 5 fit reasonably well to *C*_*z*_(*r*,t* = 88 h) (Fig. 3B; Fig. S1B). This suggests that the spatial patterns in the biofilms formed *in vitro* by wild-type NTHI possess different fractal structures extending to large (*r* > 10 μm) length scales.

### Agent-based model simulations. (i) Kinetic Monte Carlo simulation.

We implemented a kinetic Monte Carlo (kMC) scheme to simulate NTHI biofilm growth, using the rules described in Table 1. kMC methods have been used in simulating stochastic kinetics in diverse spatially resolved systems. We followed the algorithm described in reference 25. Each MC step (MCS) involved carrying out *N*_*X*_ × *N*_*Z*_ MC trials, where *N*_*X*_ × *N*_*Z*_ is the total number of compartments considered in the model. Execution of an MCS evolved the system by a time step of Δ*t*/*n*, where *n* (= 9) is the total number of rules. At each MC trial, the following steps were implemented. All the random numbers used in the following steps were drawn from a uniform distribution with the specified range.

(1) Draw two random integers, 1 ≤ λ_*x*_ ≤ *N*_*X*_, and, 1 ≤ λ_*z*_ ≤ *N*_*Z*_, to choose the compartment at the location (λ_*x*_,λ_*z*_) for execution of a rule to be decided in the next step.

(2) Call a random number λ_1_ between 0 and *n*. When *i* − 1 < *λ*_1_ ≤ *i* (*i* ≤ *n*), the process given by rule no. i is implemented with rate *k*_*i*_ by following step 3.

(3) Draw a random number λ_2_ between 0 and 1, and if λ_2_ ≤ *p*_*i*_, then process or rule 1 is implemented. *p*_*i*_ is the probability for the *i*th process to occur and is given by the equation *k*_*i*_ × Δ*t*, where *k*_*i*_ is the rate for process *i*.

(4) Go back to step 1.

### (ii) Boundary conditions.

We imposed periodic boundary conditions at *x* = 0 and *x* = *N*_*x*_ and no-flux fixed boundary conditions at *z* = 0 and *z* = *N_z_*.

### Determination of the biofilm surface and calculation of *h*_avg_ and the coefficient of variation. (i) Confocal image data.

The two-dimensional biofilm surface *z*(*x*,*y*) was determined by approaching the biofilm from the top along the *z* axis and identifying the voxel located at {*x*, *y*, *z*(*x*,*y*)} where the intensity crossed a threshold for the first time. The threshold intensity was taken to be 1/10 the highest voxel intensity in an image. The average height *h*_avg_ in the confocal images was calculated using the formula *h*_avg_ = 1/*N*_voxel_ ∑_i_ *z*_*i*_(*x*_*i*_,*y*_*i*_), where *z*_*i*_(*x*_*i*_,*y*_*i*_) denotes the biofilm height at voxel *i* on the biofilm surface and *N*_voxel_ is the total number of voxels in the computed biofilm surface. The standard deviation σ is calculated using the equation σ^2^ = 1/*N*_voxel_ ∑_*i*_ [*z*_*i*_(*x*_*i*_,*y*_*i*_)]^2^ − (*h*_avg_)^2^ and the coefficient of variation is calculated as σ/*h*_avg_.

### (ii) *In silico* model.

The same procedure as that used for the confocal imaging data was followed for the *in silico* model. In this case, the biofilm surface, *z*(*x*), resides in one dimension. The threshold is chosen to be 1 NTHI bacterial cell per *in silico* compartment.

### Evaluation of the θ exponent.

*C*(*r*,*t*) versus *r* data for *r* values of ≤1.5 μm or *r*/ξ(*t*) values of <1 were fitted with a function, 1 − *ar*^θ^, where the fitting parameters *a* and θ were determined using the Levenberg-Marquardt algorithm (45). The calculations were performed using MatLab.

### Formation of NTHI biofilms *in vitro.*

Several microbial *in vitro* biofilm models have been developed in order to study biofilm formation, structure, and physiology under specific laboratory conditions (1, 2). We generated biofilms formed *in vitro* by nontypeable *Haemophilus influenzae* strain 86-028NP or its isogenic Δ*pilA* or Δ*comE* mutants on eight-well chambered coverglasses under static conditions according to a method described previously by Jurcisek et al. (51). Briefly, bacteria from an overnight chocolate agar culture were suspended in brain heart infusion broth that had been supplemented with 2 µg/ml each of heme (Sigma-Aldrich, St. Louis, MO) and β-nicotinamide adenine dinucleotide (Fisher Scientific, Pittsburgh, PA) (sBHI) to an optical density at 490 nm of 0.65. The bacterial suspension was diluted 1:6 in medium and incubated for 3 h at 37°C, 5% CO_2_, under static conditions. The culture was further diluted 1:2,500 with sBHI, and 200 µl of the diluted bacterial suspension, equivalent to 4e4 CFU NTHI, were inoculated into each well of an eight-well chambered coverglass (Fisher Scientific). Biofilms were grown under static conditions at 37°C, 5% CO_2_ in a humidified environment. The medium was replaced with fresh sBHI at intervals of 16 h (night) and 8 h (day). Biofilms were processed for imaging at 16, 40, 64, and 88 h postseeding.

### Biofilm staining and imaging.

At the final time points, biofilms were washed twice with sterile saline solution and stained using the Live/Dead BacLight bacterial viability kit (Molecular Probes, Eugene, OR) according to the manufacturer’s instructions. Biofilms were then fixed as described previously (8). *z*-stack images were collected with a Zeiss 510 confocal laser scanning microscope (CLSM; Carl Zeiss, Thornwood, NY) with a 63×, 1.2 numerical aperture objective. All biofilm assays were performed in duplicate for a minimum of three times.

10.1128/mBio.01466-17.8 FIG S7  Results from an alternate *in silico* model for NTHI biofilm formation, where the dispersion rate increased with biofilm height. The dispersion rate increase in the model was ∝ *z*/(10 + *z*), where *z* is the distance of a compartment from the substrate at *z* = 0. (A) Variation of *C*_*z*_(*r*,t*)/*C*_*z*_(0,t*) with *r*/ξ_*z*_(t*) at larger distances for multiple *z* planes (16 μm, 19 μm, and 22 μm) for the *in silico* biofilm at t* = 88 h. The data show an absence of scaling, which is in agreement with the *in vitro* data (Fig. 3B). (B) Values of the θ exponent on different *z* planes, calculated from *C*_*z*_(*r*,t*)/*C*_*z*_(0,t*) at an *r* value of ≤5 μm. The values indicate θ < 1. These results are qualitatively similar to those for the *in silico* model investigated. Download FIG S7, PDF file, 0.5 MB. Copyright © 2017 Das et al. 2017 Das et al. This content is distributed under the terms of the Creative Commons Attribution 4.0 International license.
