# Supplementary figures and images for: Extracellular DNA and Type IV Pilus Expression Regulate the Structure and Kinetics of Biofilm Formation by Nontypeable Haemophilus influenzae
Source: mBio. 2017 Dec 19;8(6):e01466-17. doi: 10.1128/mBio.01466-17 (PMC5736908; doi:10.1128/mBio.01466-17)

**A**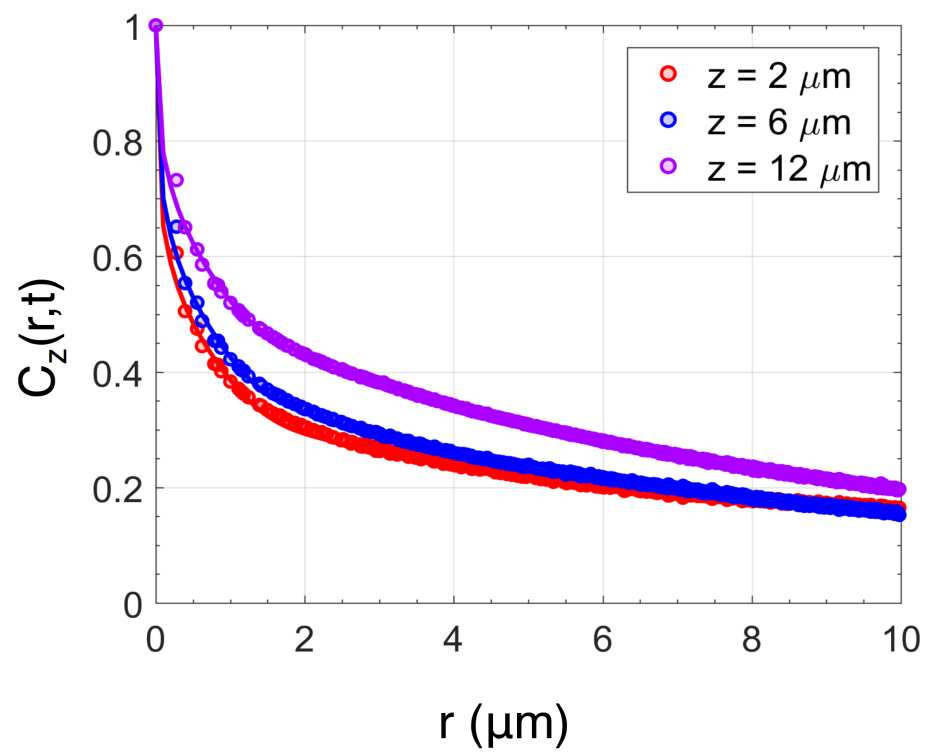**B**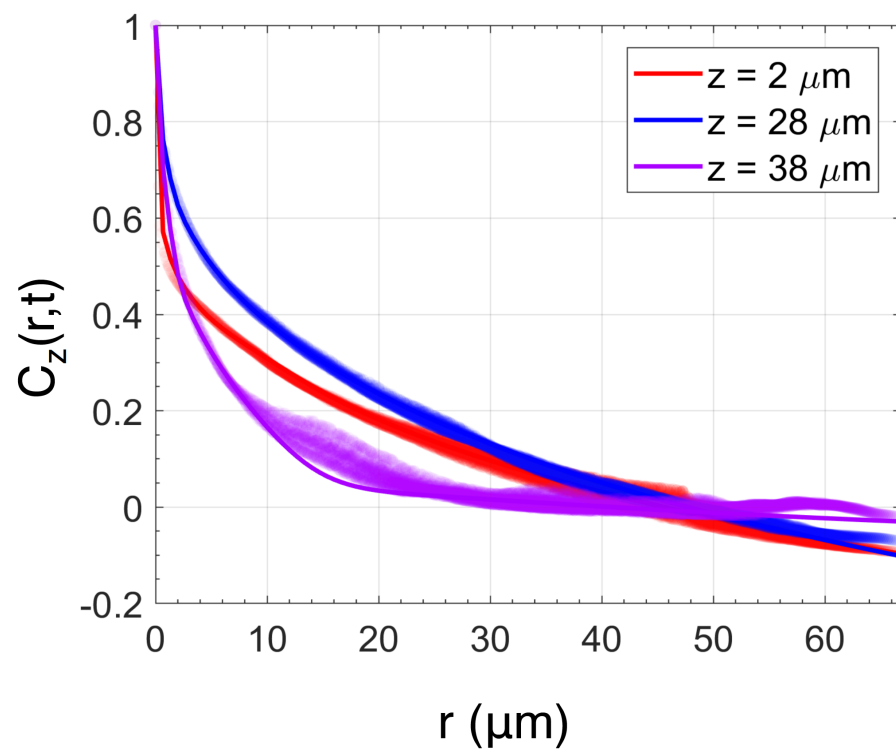

Supplement: FIG S1 [file mbo006173633sf1.pdf]

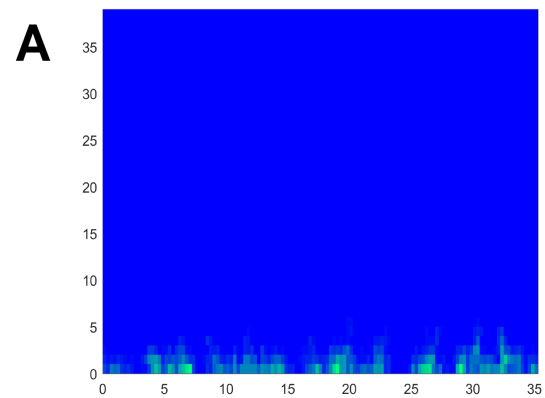

t=16h

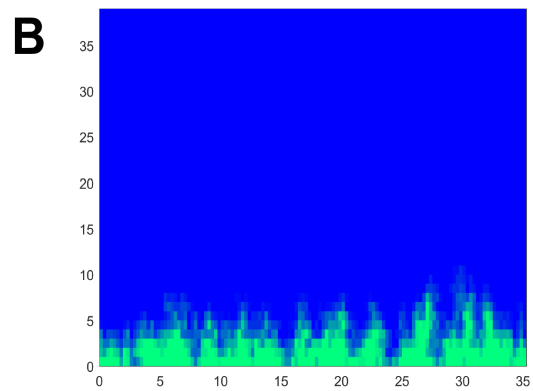

t=40h

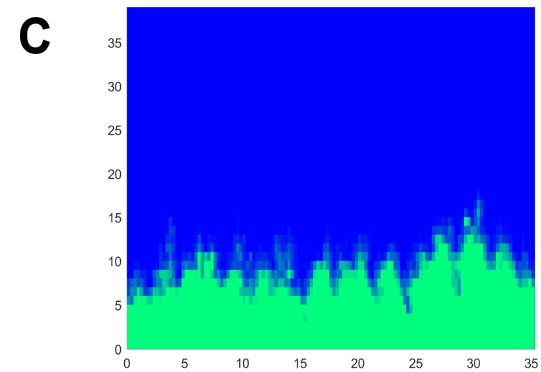

t=88h

Supplement: FIG S2 [file mbo006173633sf2.pdf]

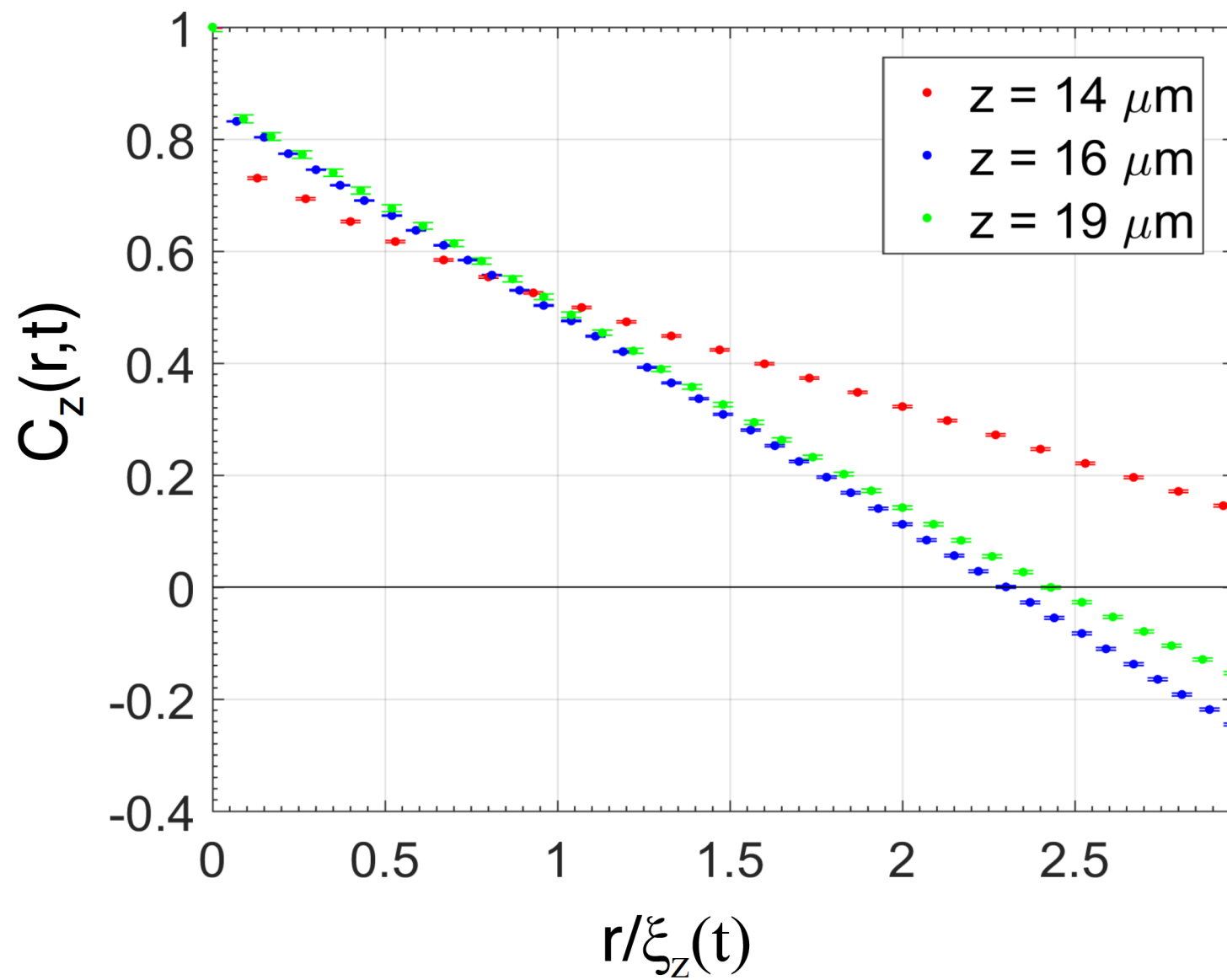

Supplement: FIG S3 [file mbo006173633sf3.pdf]

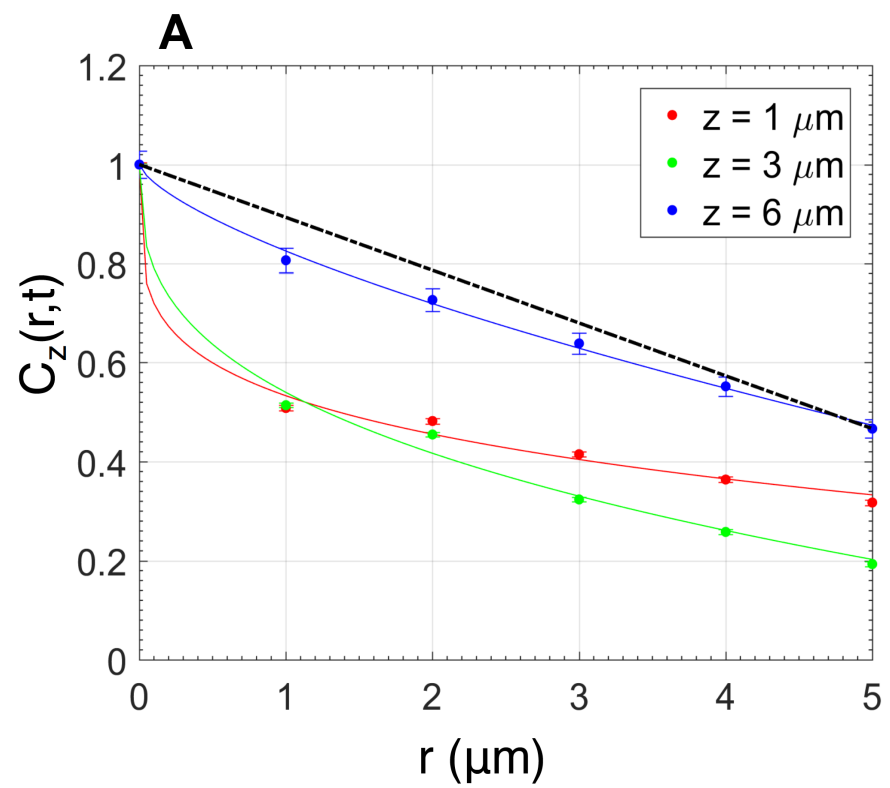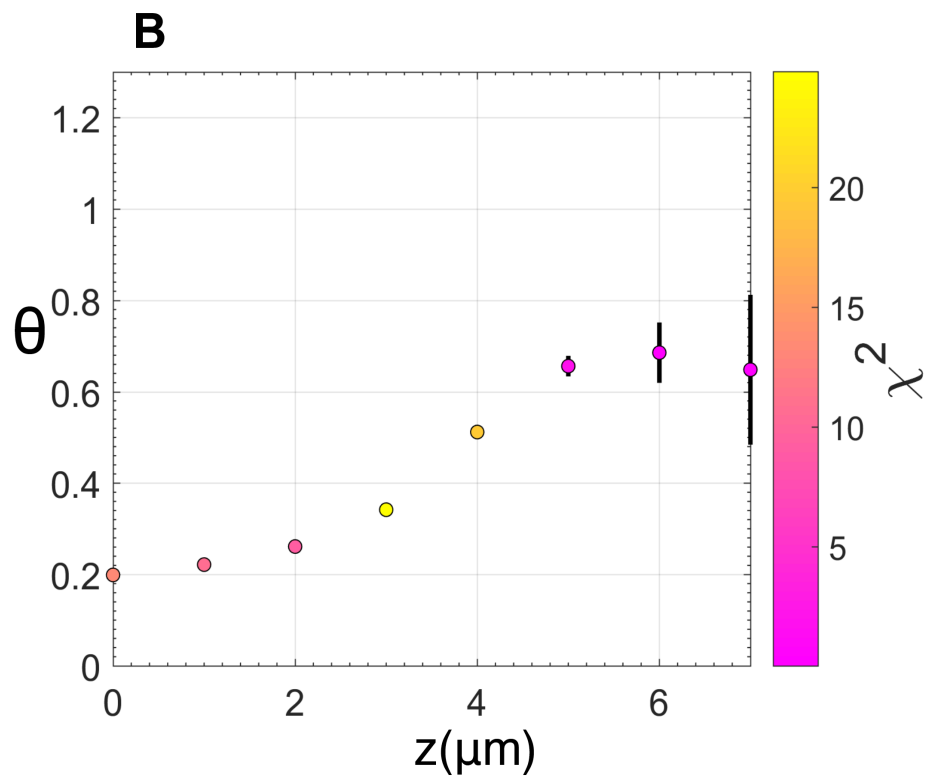

Supplement: FIG S4 [file mbo006173633sf4.pdf]

**A**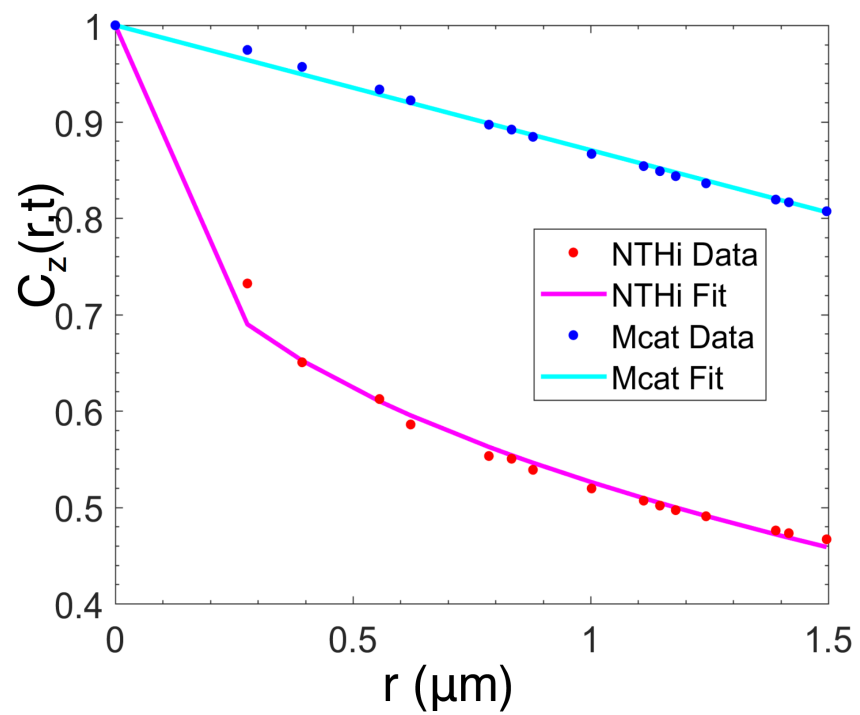**B**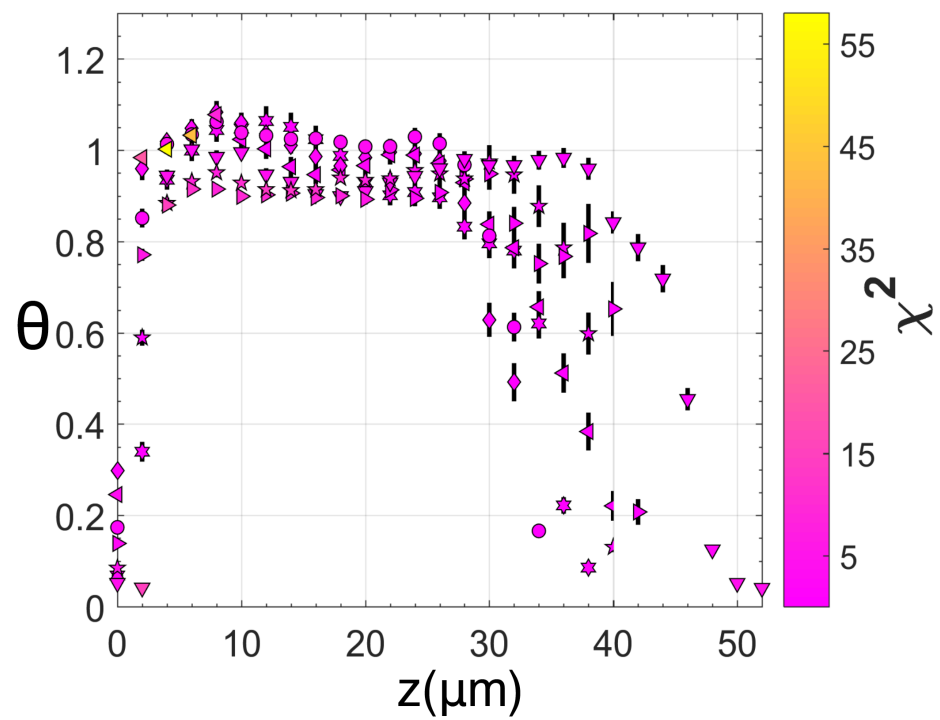

Supplement: FIG S5 [file mbo006173633sf5.pdf]

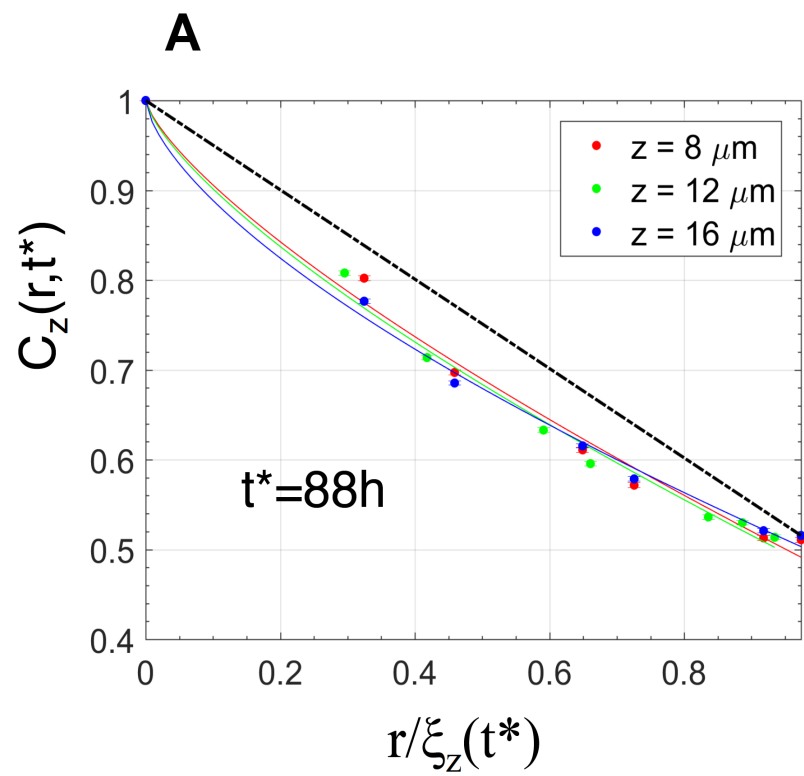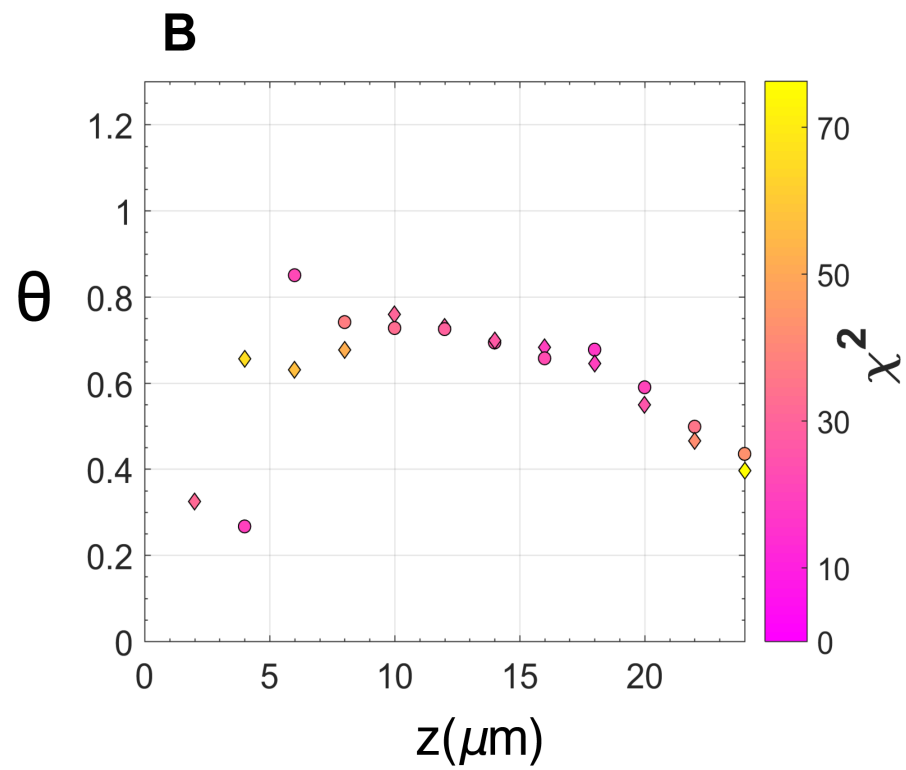

Supplement: FIG S6 [file mbo006173633sf6.pdf]

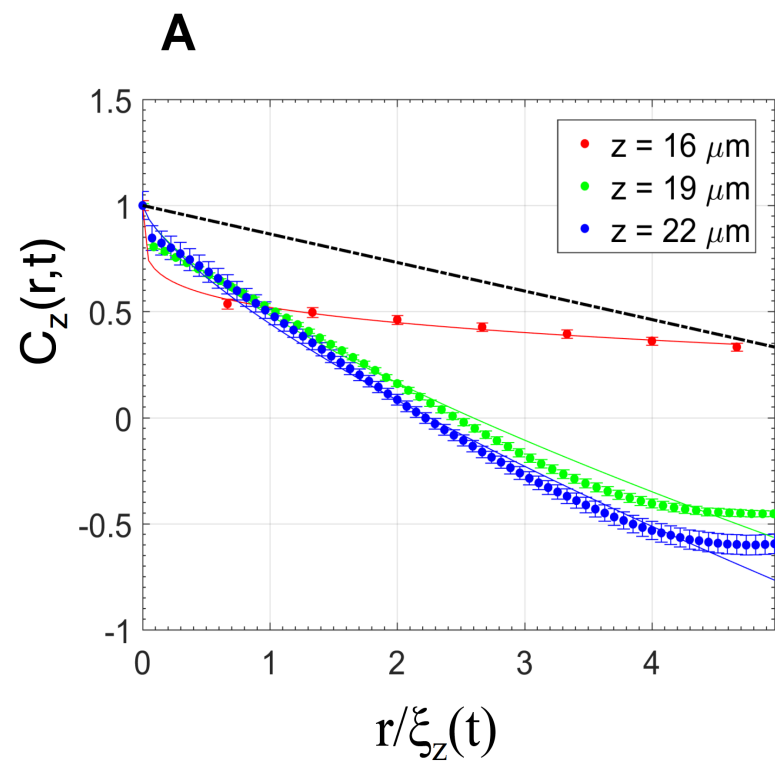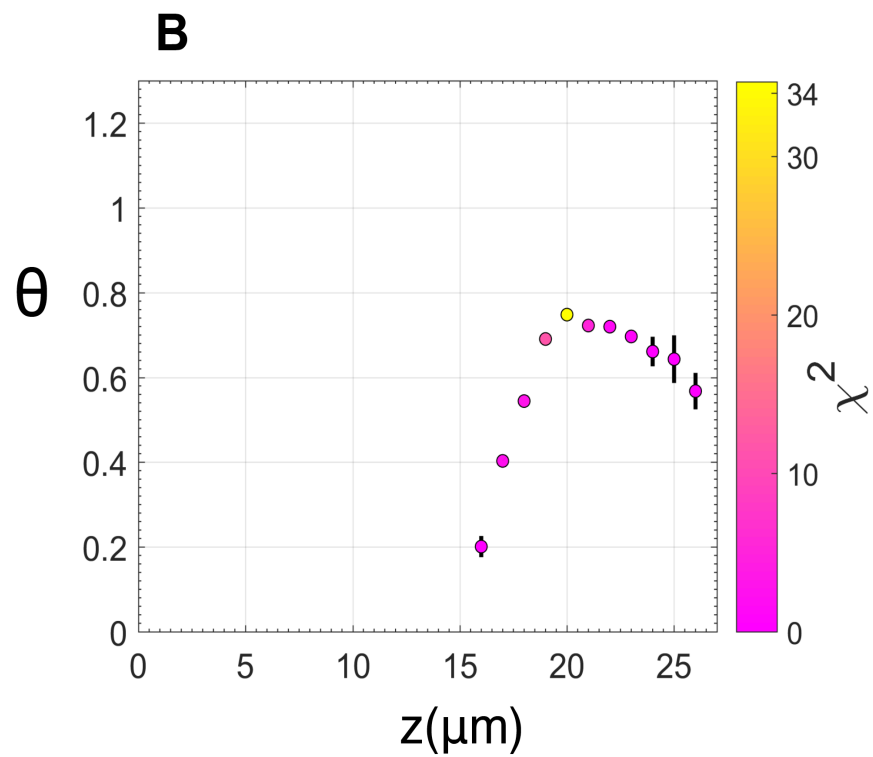

Supplement: FIG S7 [file mbo006173633sf7.pdf]
