# Supplementary material for: Extracellular DNA and Type IV Pilus Expression Regulate the Structure and Kinetics of Biofilm Formation by Nontypeable Haemophilus influenzae
Source: mBio. 2017 Dec 19;8(6):e01466-17. doi: 10.1128/mBio.01466-17 (PMC5736908; doi:10.1128/mBio.01466-17)
Supplement: TEXT S1 [file mbo006173633s1.pdf]

Calculation of the parameters used in the *in silico* model

A. Size of eDNA.

We estimated the size of eDNA secreted by NTHI from the size of the NTHI genome.

NTHI genome size is about 1,850,897 bp $\approx 1.8 \times 10^6$ bps(1). Length (l) of a base pair = 3.4A = 0.34nm=0.34×10^{-3} µm. Therefore, length (L) of the NTHI DNA is $L=1.8\times 10^6\times l=1.8\times 10^6 \times0.34\times10^{-3}$ µm = 0.612×10^3 µm=612µm.

However, the DNA will remain coiled due to entropic forces like a polymer chain. The distance between the monomers in this case is the persistence length of the DNA, which is $l_0 \approx$ 50nm = 0.05 µm(2). Thus, the DNA of length 612µm can be approximated by a Gaussian polymer with a radius of gyration(3), $(R_g)_{DNA} = (1/6\ Ll_0)^{1/2} = (612/6\times 0.05)^{1/2}$ µm $\approx 5.5/\sqrt{6} \approx 2.258$ µm. [Using $R_g^2=1/6\ Nl_0^2$, where, N is the number of monomers, $Nl_0=L$].

R_g can be used as a size estimate for the eDNA. If the whole NTHI DNA is broken into y pieces, average R_g for eDNA will be $(R_g)_{eDNA} =(R_g)_{DNA}/\sqrt{y} = 2.258/\sqrt{y}$ µm. We assume y=100, thus, $(R_g)_{eDNA} = 0.2358$ µm.

B. eDNA diffusion constant.

Diffusion of DNA strands in water is given by an empirical relation, $D_{DNA} \sim 4.9 \times 10^{-6}$ cm^2/s $\times$ [# of bps]$^{-0.72}$ (4).

Using bacterial DNA ~1800 kbps, $D_{DNA} \sim 5 \times 10^{-6}$ cm^2/s x $(1800000)^{-0.72} \approx 15.65 \times 10^{-11}$ cm^2/s = 1.5×10^{-10} cm^2/s = 1.5×10^{-10} (10^8) µm^2/s = 0.015 µm^2/s = 0.9 µm^2/min $\approx$ 1.0 µm^2/min. If the eDNA is broken into y pieces, $(D)_{eDNA}=(D)_{DNA}\times (y)^{0.72}$. When y=100, $(D_e)_{whole}\times27.5 \approx 27.5$ µm^2/min. However, the diffusion constant of molecules within the biofilm is 0.2-0.8 times smaller compared to that in the aqueous medium (5). Thus, $(D)_{eDNA}$ in the biofilm will range between 5.5-22.0 µm^2/min. We chose a value of 10 µm^2/min for the eDNA diffusion constant in the simulation.

C. Calculation of the planktonic NTHI diffusion.

We used Stokes-Einstein relationship (3) to calculate the diffusion constant for an NTHI particle in water: $D_{NTHI}=k_BT/(6\pi\eta r)$, where, $k_B \equiv$ Boltzmann's constant=1.38×10^{-16} erg/K°, T$\equiv$ solution temperature = 310° K (37° C), $\eta \equiv$ viscosity of water = 6.9×10^{-3} dyn.s/cm^2 (at 37° C), r$\equiv$ radius of the sphere $\approx$ 0.5 µm for NTHI particle.

Thus, $D_{NTHI}=1.38\times 310\times 10^{-16}/(6\pi \times 6.9 \times 10^{-3}\times 0.5 \times 10^{-4}) = 427/65.03\times 10^{-9}$ cm^2/s=6.5×10^{-1} µm^2/s=39.0 µm^2/min. Since the diffusion constant of the NTHI will be smaller in the biofilm by 0.2-0.8 fold compared to that in the aqueous medium(5), D_{NTHI} in the biofilm will range between 7.8-31.2 µm^2/min. We chose a value of 10 µm^2/min for the D_{NTHI} in the simulation.

D. Calculation of eDNA production rate.

Following Ref. (6) eDNA production saturates after 72 hrs. Between 16 to 24 hrs the eDNA content (x) in a culture well increased from 700ng to about 1300ng. First, we calculate the density of the eDNA in the biofilm from the measured weight.

300-700ng of eDNA was recovered in a chamber slide in Ref. (6).

1kbp DNA $\approx 10^{-6}$ pg.
1pg = 10^{-3} ng.

Therefore, 100ng of NTHI DNA should contain 10^5pg $\approx 10^{11}$ kbp DNA.
10^{11} kbp DNA $\approx 5.5\times 10^6$ molecules of NTHI DNA.

The base of a single biofilm culture chamber has an area $\approx$ 10mm $\times$ 10mm = 100×10^6 $(\mu m)^2$, and, approximating that the bacterial DNA is homogenized in the biofilm (height of ~20μm). Thus the biofilm volume is $100\times20\times10^6$ $(\mu m)^3=2.0\times10^9(\mu m)^3$ and the density for 100ng of bacterial DNA will be 5.5×10^6 molecules/$(2\times10^9(\mu m)^3)$ = 2.25×10^{-3} molecules/$(\mu m)^3$. If the DNA is broken into y(=100) eDNA fragments, the density of 100ng of eDNA will be $2.25\times 10^{-3}\times$ y molecules/$(\mu m)^3$.

A constant growth rate fit (dx/dt=k) to that data generate an estimate for the production rate, k, given by, $k=[x(t+\Delta t)-x(t)]/\Delta t$ = 600ng/8hrs=$6\times0.00225\times$y molecule/$(\mu m)^3$/(8×60mins)= $2.81\times 10^{-5}\times$ y molecule/$(\mu m)^3$/mins=0.0000281y molecule/($(\mu m)^3$mins). We assume y=100, thus, k=600ng/8hrs =0.00281 molecule/($(\mu m)^3$mins)$\approx$ 0.003 molecule/($(\mu m)^3$mins). Between 24-36hrs the rate is, 200ng/8hrs = 0.00094 molecule/($(\mu m)^3$mins). We used a constant rate for k = 0.003 molecule/($(\mu m)^3$mins in the simulation.

E. Calculation of NTHI density.
Maximum packing fraction of cylinders (aspect ratio 1.2) packed randomly is $\approx$ 0.66 (7). Assuming NTHI is a cylinder of length 1.0 μm and a diameter 0.33 μm, the volume of an NTHI bacterium is $v_0 = \pi\ (0.33/2)^2\ (1.0)\ (\mu m)^3 = 0.0855(\mu m)^3$. Thus, in a volume of l_0^3, the maximum the number of NTHI particles is, $n_{max} = l_0^3/v_0 = l_0^3/\ 0.0855$. For, $l_0 = 1$ μm, n_{max}=11.7. Using the packing fraction, n_{max}= 0.66 × 11=7.722. We used, n_{max}=7 in the simulation.

1. Mell JC, Sinha S, Balashov S, Viadas C, Grassa CJ, Ehrlich GD, Nislow C, Redfield RJ, Garmendia J. 2014. Complete Genome Sequence of Haemophilus influenzae Strain 375 from the Middle Ear of a Pediatric Patient with Otitis Media. Genome Announc 2.

2. Smith SB, Finzi L, Bustamante C. 1992. Direct mechanical measurements of the elasticity of single DNA molecules by using magnetic beads. Science 258:1122-6.
3. Doi M, Edwards SF. The theory of polymer dynamics.
4. Lukacs GL, Haggie P, Seksek O, Lechardeur D, Freedman N, Verkman AS. 2000. Size-dependent DNA mobility in cytoplasm and nucleus. J Biol Chem 275:1625-9.
5. Stewart PS. 2003. Diffusion in biofilms. J Bacteriol 185:1485-91.
6. Jones EA, McGillivary G, Bakaletz LO. 2013. Extracellular DNA within a Nontypeable Haemophilus influenzae -Induced Biofilm Binds Human Beta Defensin-3 and Reduces Its Antimicrobial Activity. J Innate Immun 5:24-38.
7. Zhang W. 2006. EXPERIMENTAL AND COMPUTATIONAL ANALYSIS OF RANDOM CYLINDER PACKINGS WITH APPLICATIONS. PhD. Louisiana State University and Agricultural and Mechanical College.
